# Synthesis of the first examples of iminosugar clusters based on cyclopeptoid cores

**DOI:** 10.3762/bjoc.10.144

**Published:** 2014-06-23

**Authors:** Mathieu L Lepage, Alessandra Meli, Anne Bodlenner, Céline Tarnus, Francesco De Riccardis, Irene Izzo, Philippe Compain

**Affiliations:** 1 Laboratoire de Synthèse Organique et Molécules Bioactives (SYBIO), Université de Strasbourg/CNRS (UMR 7509), Ecole Européenne de Chimie, Polymères et Matériaux, 25 rue Becquerel, 67087 Strasbourg, France; 2 Department of Chemistry and Biology, University of Salerno, Via Giovanni Paolo II, 132, I-84084 Fisciano Salerno, Italy; 3 Université de Haute Alsace, Laboratoire de Chimie Organique et Bioorganique, EA4466, ENSCMu, 3, rue Alfred Werner, 68093 Mulhouse Cedex, France; 4 Institut Universitaire de France, 103 Bd Saint-Michel, 75005 Paris, France

**Keywords:** cyclopeptoids, glycosidases, iminosugars, inhibitors, multivalency, mutivalent glycosystems

## Abstract

Cyclic *N*-propargyl α-peptoids of various sizes were prepared by way of macrocyclizations of linear *N*-substituted oligoglycines. These compounds were used as molecular platforms to synthesize a series of iminosugar clusters with different valency and alkyl spacer lengths by means of Cu(I)-catalysed azide–alkyne cycloadditions. Evaluation of these compounds as α-mannosidase inhibitors led to significant multivalent effects and further demonstrated the decisive influence of scaffold rigidity on binding affinity enhancements.

## Introduction

**Figure 1:**
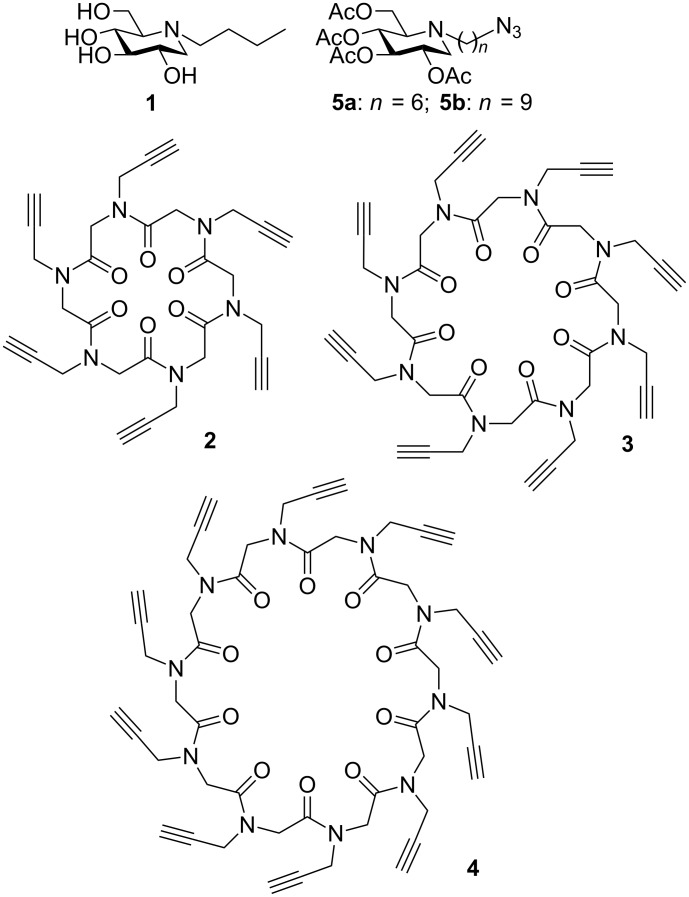
*N*-Bu-DNJ (**1**), azide-armed DNJ derivatives **5** and cyclopeptoid scaffolds **2**–**4**.

Within a few years, the field of multivalent glycosidase inhibitors has witnessed tremendous advancement. Since the report in 2009 of the first quantifiable multivalent effect in glycosidase inhibition [1–2], the pace of progress has been breath-taking with the discovery of iminosugar clusters showing outstanding affinity enhancements of up to four orders of magnitude over the parent monovalent analogues [3–7]. The best results were obtained with multivalent systems based on C_60_ [3], β-cyclodextrin [4–5] and porphyrin [7] cores, and with nanoparticles prepared by self-assembly of iminosugar-based glycopolypetides [6]. So far, the largest multivalent effect (up to 610-fold relative inhibition potency increase on a valency-corrected basis) has been achieved on jack bean α-mannosidase with β-cyclodextrin-based analogues displaying 14 copies of 1-deoxynojirimycin (DNJ) [4]. Applications of the inhibitory multivalent effect to glycosidases of therapeutic interest were promptly performed and promising results were obtained in the field of Gaucher disease, the most common lysosomal storage disorder [8–9]. In 2013, the first description of a multivalent effect for correcting protein folding defects in cells was reported with trivalent DNJ clusters [10]. These compounds were found to overcome the processing defect of the mutant CFTR protein involved in cystic fibrosis, and to be up to 2 orders of magnitude more efficient as CFTR correctors than the clinical candidate miglustat (*N*-Bu-DNJ, **1**, Figure 1).

The mechanisms underlying the inhibitory multivalent effect were studied with different methods such as isothermal titration calorimetry, competitive lectin-enzyme assays, X-ray crystallography or atomic force spectroscopy [7,11–13]. At this stage of research, one of the main challenges in the field is to design optimal systems that not only display large multivalent effects but also possess the desired properties for particular applications. In this context, the choice of the scaffold is crucial as it defines the valency, the size and the shape of the multivalent architectures. Due to their broad chemical diversity, rapid and convenient synthetic access, improved proteolytic stability and cell permeability over peptides, *N*-substituted glycine oligomers, called peptoids [14–17], appear as promising scaffolds for the synthesis of glycoconjugates of biological interest [14–18]. Combination of these advantages has led to many examples of biologically active peptoids [19–21]. So far, some syntheses of *N*-, *O*-, *C*- and *S*-linked glycopeptoids have been reported [22–31] and few of them are related to cyclopeptoids [32–33]. One of the most intriguing features of peptoids is their capacity to generate cyclic structures, which can expand the utility of this platform to multivalent chemical achitectures [34]. Conformation, size, charge and branching of these cyclic scaffolds influence the pharmacological profile of the products [35–39]. Moreover, macrocyclization enforces the rigidity of the more flexible linear peptoid skeleton and generally produces enhancement in biological activities [21,37]. In this context cyclopeptoids **2**–**4** appear as ideal building blocks because of their simplicity of synthesis and easy functionalization by click reaction (Figure 1). In the present paper, we report the synthesis of the first examples of cyclopeptoid-based iminosugar clusters. The influence of valency, size, linker and scaffold structure on jack bean α-mannosidase inhibition was evaluated with a series of 6- to 10-valent DNJ derivatives with two different alkyl spacer lengths (C_6_ or C_9_).

## Results and Discussion

Our synthetic strategy was based on a convergent approach involving the attachment of azide-armed iminosugars **5** onto polyalkyne “clickable” scaffolds **2**–**4** by Cu(I)-catalyzed azide–alkyne cycloaddition (CuAAC) reactions [40–41] (Figure 1). *N*-alkyl derivatives of DNJ were logically chosen as the peripheral ligands because of the therapeutic relevance of these compounds [42]. In addition, most of the glycosidase inhibitor clusters published in the literature are based on these binding motifs [1–7,11,43] providing thus the opportunity to assess the relevance of cyclopeptoid cores by comparison with the other platforms already described.

### Scaffold synthesis

**Scheme 1:**
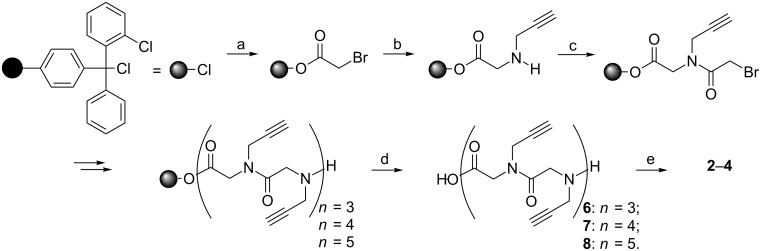
Sub-monomer approach for the synthesis of cyclopeptoids **2**–**4**: DIPEA = *N*,*N*-diisopropylethylamine; DIC = *N*,*N*’-diisopropylcarbodiimide; HATU *O*-(7-azabenzotriazole-1-yl)-*N,N,N′,N′-*tetramethyluronium hexafluorophosphate. (a) bromoacetic acid, DIPEA, CH_2_Cl_2_; (b) propargylamine (10 equiv), DMF; (c) bromoacetic acid, DIC, DMF; (d) CH_2_Cl_2_/HFIP (4:1); (e) HATU, DIPEA, DMF.

The linear precursors of cyclic scaffolds (**2**–**4,** Figure 1) were prepared using the sub-monomer approach developed by Zuckerman et al. [44] through a two-step sequence, repeated iteratively, to obtain the desired oligomers (Scheme 1). Each monomer is constructed on the 2-chlorotrityl resin from *C*- to *N*-terminus using *N*,*N’*-diisopropylcarbodiimide (DIC)-mediated acylation with bromoacetic acid, followed by amination with the propargyl amine. After the completion of synthesis, the oligomers were cleaved from the resin using a 4:1 solution of CH_2_Cl_2_/hexafluoroisopropanol (HFIP). Macrocyclizations of the linear *N*-substituted oligoglycines **6**–**8** proceeded smoothly giving, under high dilution conditions (3.0 × 10^−3^ M) and in the presence of the efficient coupling agent HATU, the desired cyclic peptoids **2**–**4** (Scheme 1). After purification, compounds **2**–**4** were recovered in 31%, 32% and 12% overall yield respectively.

### DNJ cluster synthesis

**Scheme 2:**
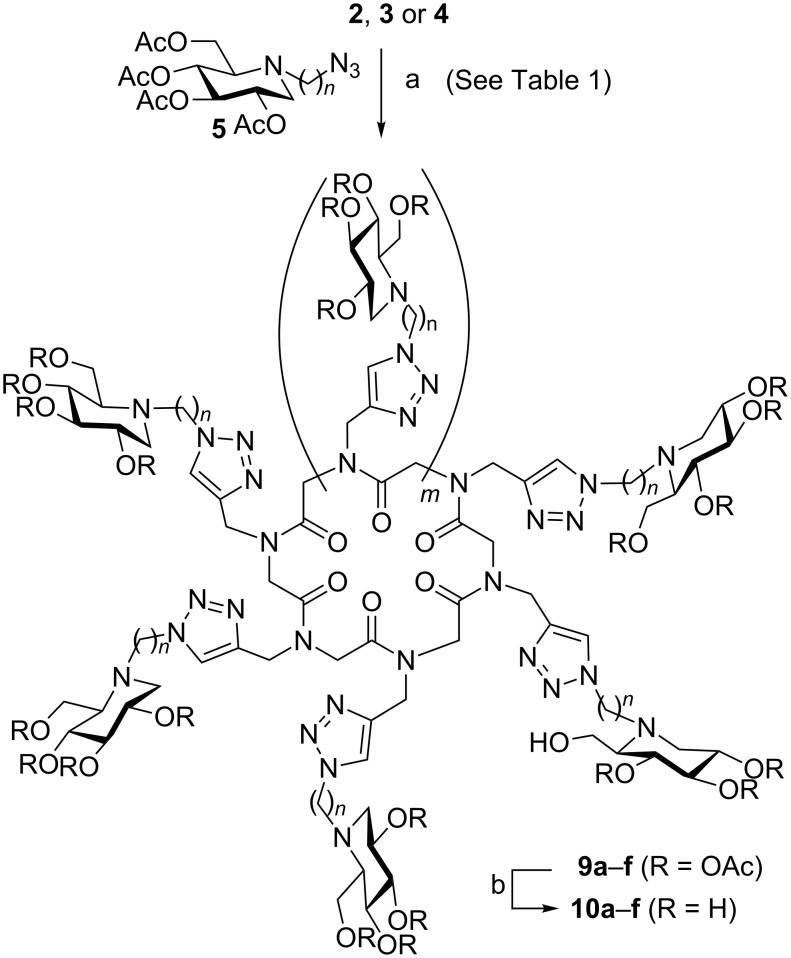
Synthesis of DNJ clusters **10**: (a) CuSO_4_·5H_2_O cat., sodium ascorbate, DMF/H_2_O (5:1), MW, 80 °C; (b) Amberlite IRA 400 (OH^–^), MeOH/H_2_O (1:1), 40 °C. Overall yields from compounds **2**, **3** or **4**: **10a** (*n* = 6, *m* = 1) 95%; **10b** (*n* = 9, *m* = 1) 83%; **10c** (*n* = 6, *m* = 3) 69%; **10d** (*n* = 9, *m* = 3) 80%; **10e** (*n* = 6, *m* = 5) 70%; **10f** (*n* = 9, *m* = 5) 80%.

The last stages of the DNJ cluster synthesis were based on a robust two-step process, recently developed in our group for the preparation of iminosugar click clusters [4–5,9–11]. The first step of the process involved the attachment of peracetylated azido iminosugars **5** [4] onto polyalkyne scaffolds **2**–**4** by microwave-assisted CuAAC reaction (Scheme 2).

**Figure 2:**
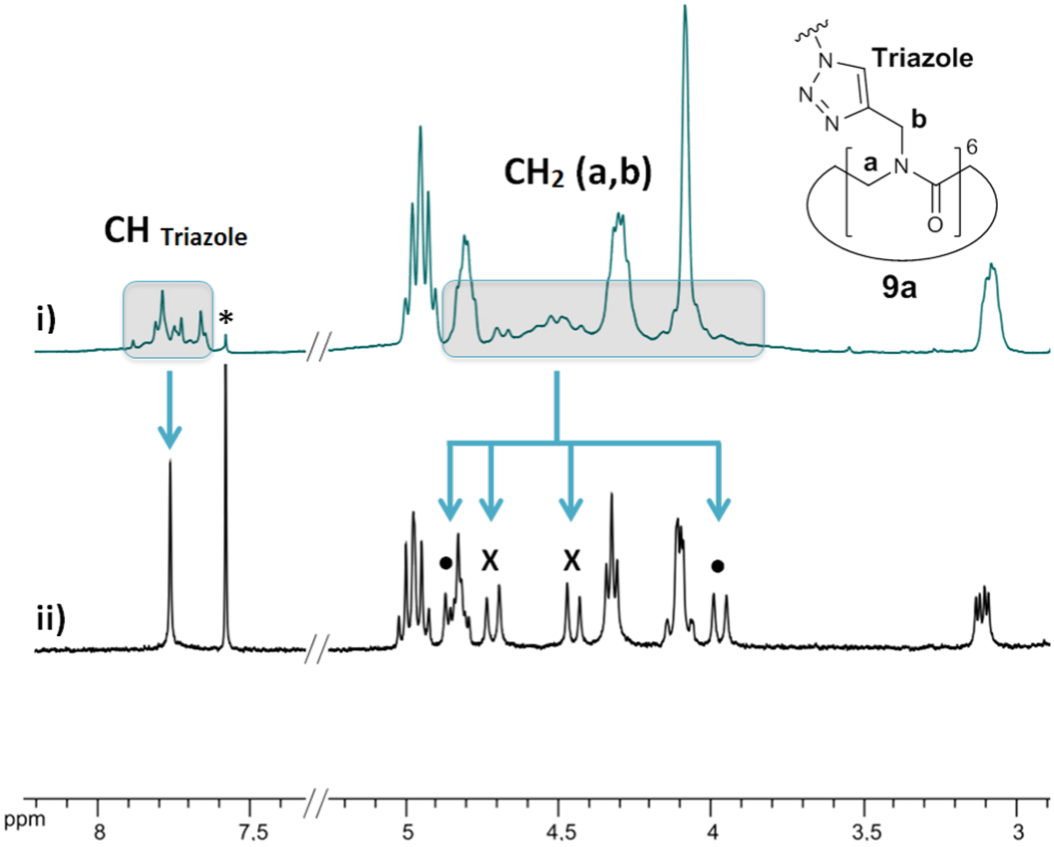
i) Partial ^1^H NMR spectrum (400 MHz, CD_3_CN/CDCl_3_ 9:1) of compound **9a**; ii) Partial ^1^H NMR spectrum (400 MHz, CD_3_CN/CDCl_3_ 9:1) of compound **9a** with sodium picrate (11 equiv). * Residual solvent peak for CDCl_3_. • and X are assigned to protons a or b.

The multiconjugation reaction proceeded smoothly to afford the six desired DNJ clusters **9** in 69–95% yields. With the exception of octavalent iminosugars **9c** (*n* = 6) and **9d** (*n* = 9), these compounds showed complex ^1^H NMR spectra at room temperature as exemplified by compound **9a** (Figure 2i). This phenomenon, already observed for *N*-substituted cyclic α-peptoid derivatives [35–39], indicated the presence of more than one conformer in slow exchange on the NMR time scale. It is well known that the conformational heterogeneity is due to tertiary amide bonds, which can isomerize more readily than secondary amides, and to the absence of amide protons, which stabilize secondary structure by backbone hydrogen bonding [15–16]. As we have previousy demonstrated, this heterogeneity can be reduced by metal chelation [35,38]. Addition of an excess of sodium picrate to **9a** indeed dramatically simplified the ^1^H NMR spectrum by inducing the formation of a sodium complex with a 6-fold symmetry (Figure 2ii).

Subsequent *O*-deacetylation of compounds **9** using anion exchange Amberlite IRA-400 (OH^−^) resin provided the final deprotected iminosugar clusters **10** in high yields without affecting the potentially labile amide bond.

**Figure 3:**
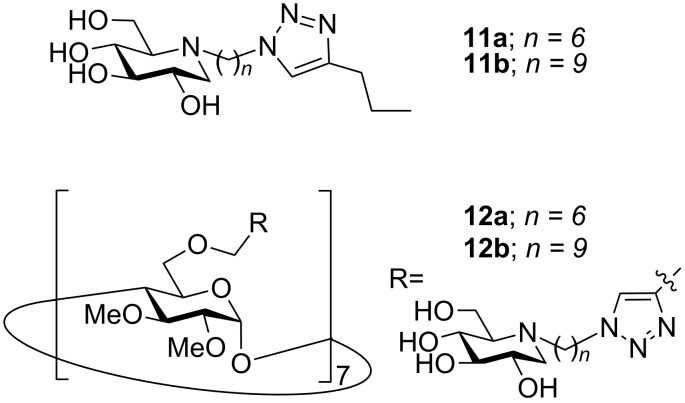
Monovalent models **11** and 7-valent DNJ derivatives **12**.

As indicated in the introduction, the best multivalent effects in glycosidase inhibition observed so far were obtained with jack bean α-mannosidase [1–7,11–12]. Accordingly, in order to complete these compelling investigations, evaluation of the inhibition potency of multivalent iminosugars **10** was performed on this peculiar enzyme (Table 1). Related monovalent controls **11** [3–4] as well as 7-valent β-cyclodextrin-based DNJ clusters **12** [4] have been included for comparative purposes (Figure 3). Our results clearly point out that all cyclopeptoid-based clusters **10** display a significant multivalent effect (*rp*/*n* > 1), with 6-valent iminosugar **10a** as a single exception.

**Table 1:** Relative inhibition potency of cyclopeptoid-based clusters **10** and inhibitory activity (*K*_i_, μM) against jack bean α-mannosidase.

Compound	Valency	Linker length	*K*_i_ (μM)	*rp*^a^	*rp*/*n*^b^

**11a**	1	C_6_	322 [3–4]	–	–
**10a**	6	C_6_	65 ± 24	4.9	0.8
**10c**	8	C_6_	21 ± 2	15	1.9
**10e**	10	C_6_	15 ± 10^c^	21	2.1
**12a**	7	C_6_	7.7 [4]	42	6.0
**11b**	1	C_9_	188 [3–4]	–	–
**10b**	6	C_9_	11 ± 1	17	2.8
**10d**	8	C_9_	8 ± 3	23	2.9
**10f**	10	C_9_	5 ± 1	38	3.8
**12b**	7	C_9_	0.36 [4]	522	75

^a^Relative inhibition potency = *K*_i_ (monovalent reference)/*K*_i_ (glycocluster). ^b^*rp*/*n* = Relative inhibition potency/number of iminosugar units. ^c^Single determination of *K*_i_ without duplicate.

Increasing the valency (from 6 to 10) or the linker length (from C_6_ to C_9_) resulted in increased inhibition potencies when compared to the corresponding monovalent models **11**, the best result being obtained with 10-valent DNJ cluster **10f** with a C_9_ linker (*rp*/*n* ~ 4). However, the binding enhancements were found to be 2- to 31-fold lower than the ones observed with the related 7-valent DNJ clusters **12** with identical alkyl spacer length but a different core (β-cyclodextrin). These results may indicate that the ligand spatial presentation in cyclopeptoid-based iminosugars **10** is not optimal to achieve a substantial multivalent effect. It has been shown recently that the use of rigid scaffolds such as porphyrin or C_60_ could lead to large multivalent effects (up to 200-fold on a valency-corrected basis) [3,7]. The modest inhibition enhancements observed with DNJ-cyclopeptoid conjugates **10** could thus be due to the high flexibility of their amide backbone [14–17].

## Conclusion

In conclusion, we have reported the efficient synthesis of the first examples of cyclopeptoid-based iminosugar clusters and their evaluation as α-mannosidase inhibitors. Modest but significant inhibitory multivalent effects were observed for most of the compounds evaluated. This study further highlights the decisive impact of the scaffold rigidity on binding affinity enhancements. In connection with our recent work in the field of rare genetic diseases [8–10], further evaluation of neoglycopeptoids in cell systems are currently underway in our laboratory. The intrinsic advantages of cylopeptoid scaffolds including improved cell permeability and proteolytic stability are indeed expected to be most beneficial for this exploratory work.

## Experimental

### General information

NMR spectra were recorded on Bruker 300, 400 and 500 MHz spectrometers with solvent peaks as reference. Carbon multiplicities were assigned by distortionless enhancement by polarization transfer (DEPT) experiments. The ^1^H signals were assigned by 2D experiments (COSY). ESI–HRMS mass spectra were carried out on a Bruker MicroTOF spectrometer. Purifications were performed with silica gel 60 (230–400 mesh, 0.040–0.063 mm).

#### General procedure for the synthesis of cyclopeptoid-based iminosugar click clusters **9a–f**

To a solution of the cyclopeptoid **2, 3** or **4** (typically 5 to 15 mg) and ligand **5a** or **5b** (1.1 equiv/alkyne moiety) in DMF (typically 0.5 to 1 mL) in a microwave vial was added a bright yellow suspension of CuSO_4_·5H_2_O (10 mol %/alkyne moiety) and sodium ascorbate (20 mol %/alkyne moiety) in water (typically 0.1 to 0.2 mL). The mixture was stirred and heated under microwave irradiation for 3 h at 80 °C. The mixture was concentrated, diluted in a 9:1:1 (v/v/v) mixture of MeCN/water/30 wt %-NH_4_OH and filtrated with the same eluent (25 mL) on a small pad of SiO_2_ (typically 1 cm thick), whose top surface became blue after copper complexation with NH_3_. The filtrate was concentrated and then filtrated on another pad of SiO_2_ (typically 1 cm wide and 2 cm thick), eluting it with AcOEt/PE 4:6 (25 mL) to recover clean unclicked ligand **5a** or **5b**, and then with MeCN/water 8:2 (25 mL) to afford iminosugar click clusters **9a–f** as pale brown translucent wax after concentration.

#### General procedure for the synthesis of deprotected cyclopeptoid-based iminosugar click clusters **10a–f**

To a solution of acetate-protected iminosugar click clusters **9a–f** in a 1:1 mixture of water/MeOH (typically 600 µL/µmol) was added Amberlite IRA400 (OH^–^) (5.5*n* g/mmol of substrate; *n* = number of acetate groups). The suspension was softly stirred overnight at 40 °C. Then the mixture was filtrated and the filtrate was concentrated to afford deprotected iminosugar click clusters **10a–f** in quantitative yields.

#### Compound **9a**


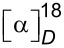
 +6.2 (*c* 1, CHCl_3_); ^1^H NMR (CD_3_CN/CDCl_3_ 9:1 + 11 equiv sodium picrate, 400 MHz) δ 7.76 (s, 6H, H-1'), 4.97 (m, *J* = 10.3 Hz, 12H, H-3, H-4), 4.85 (d, *J* = 16.3 Hz, 6H, H-3' or H-5'), 4.83 (td, *J* = 9.8, 5.3 Hz, 6H, H-2), 4.71 (d, *J* = 16.3 Hz, 6H; H-3' or H-5'), 4.45 (d, *J* = 16.3 Hz, 6H, H-3' or H-5'), 4.32 (t, *J* = 7.0 Hz, 12H, H-12), 4.10 (dd, *J* = 19.4, 13.0 Hz, 12H, H-6), 3.97 (d, *J* = 16.3 Hz, 6H, H-3' or H-5'), 3.11 (dd, *J* = 11.1, 5.3 Hz, 6H, H-1a), 2.70 (m, 6H, H-7a), 2.68 (d, *J* = 8.8 Hz, 6H, H-5), 2.51 (m, 6H, H-7b), 2.35 (dd, *J* = 12.7, 11.1 Hz, 6H, H-1b), 1.95 (s, 72H, AcO), 1.85 (m, 12H, H-11), 1.40 (m, 12H, H-8), 1.28 (m, 24H, H-9, H-10) ppm; ^13^C NMR (CD_3_CN/CDCl_3_ 9:1, 100 MHz) δ 171.4, 170.94, 170.88, 170.6, 170.6–168.9, 144.4–142.8, 124.7–123.8, 75.3, 70.5, 70.2, 62.1, 60.5, 53.4, 52.3, 50.8, 50.3–48.5, 44.9–42.6, 30.9, 27.3, 27.0, 25.2, 21.1 ppm; HRMS–ESI (*m*/*z*): [M + 2H]^2+^ calcd for C_150_H_224_N_30_O_54_ 1654.7847; found: 1654.7827.

#### Compound **10a**


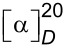
 −28.0 (*c* 0.1, H_2_O/DMSO 1:1 + 0.1% TFA); ^1^H NMR (D_2_O + 0.1% TFA, 500 MHz) δ 8.21–7.60 (m, 6H, H-1’), 5.20–3.72 (br m, 12H, H-3’ and H-5’), 4.35 (s, 12H, H-12), 3.85 (s, 12H, H-6), 3.59 (s, 6H, H-2), 3.43 (s, 6H, H-4), 3.31 (s, 6H, H-3), 3.16 (s, 6H, H-1a), 2.91 (s, 6H, H-7a), 2.78 (s, 6H, H-7b), 2.56 (s, 12H, H-1b and H-5), 1.84 (s, 12H, H-11), 1.51 (s, 12H, H-8), 1.25 (s, 24H, H-9 and H-10) ppm; ^13^C NMR (D_2_O + 0.1% TFA, 125 MHz) δ 173.4–169.3, 145.6–142.8, 127.4–124.6, 78.9, 70.4, 69.3, 66.8, 57.5, 55.9, 53.9, 51.9, 51.5–49.3, 46.3–43.3, 30.9, 27.3, 26.8, 24.0 ppm; HRMS–ESI (*m*/*z*): [M + 2H]^2+^ calcd for C_102_H_174_N_30_O_30_ 1150.6579; found: 1150.6626.

## Supporting Information

File 1 Mannosidase inhibition assay procedures, synthesis and NMR spectra of all new compounds.
