# Supplementary material for: Synthesis of the first examples of iminosugar clusters based on cyclopeptoid cores
Source: Beilstein J Org Chem. 2014 Jun 23;10:1406–12. doi: 10.3762/bjoc.10.144 (PMC4077385; doi:10.3762/bjoc.10.144)

Supporting Information

for

Synthesis of the first examples of iminosugar clusters based on cyclopeptoid cores

Mathieu L. Lepage,[1] Alessandra Meli,[2] Anne Bodlenner,[1] Céline Tarnus,[3] Francesco De Riccardis,[2] Irene Izzo*[2] and Philippe Compain*[1,4]

Address: [1]Laboratoire de Synthèse Organique et Molécules Bioactives (SYBIO), Université de Strasbourg/CNRS (UMR 7509), Ecole Européenne de Chimie, Polymères et Matériaux, 25 rue Becquerel, 67087 Strasbourg, France, [2]Department of Chemistry and Biology, University of Salerno, Via Giovanni Paolo II, 132, I-84084 Fisciano Salerno, Italy, [3]Université de Haute Alsace, Laboratoire de Chimie Organique et Bioorganique, EA4466, ENSCMu, 3, rue Alfred Werner, 68093 Mulhouse Cedex, France and [4]Institut Universitaire de France, 103 Bd Saint-Michel, 75005 Paris, France.

Email: Philippe Compain - philippe.compain@unistra.fr; Irene Izzo - iizzo@unisa.it

*Corresponding author

Mannosidase inhibition assay procedures, synthesis and NMR spectra of all new compounds

General procedure for inhibition assay

The *p*-nitrophenyl-α-D-mannopyranoside and α-mannosidase (EC 3.2.1.24, from jack bean, K_m = 2.0 mM pH 5.5) were purchased from Sigma–Aldrich. The release of *p*-nitrophenol was measured continuously at 385 nm to determine initial velocities.[1] All kinetics were performed at 35 °C and started by substrate addition in a 1 mL assay medium (acetate buffer, 0.2 M, pH = 5) containing α-mannosidase (5 mU), substrate (varying concentration from K_m/4 to K_m value) in presence or absence of various concentrations of inhibitor. K_i values were determined in duplicate, using the Dixon graphical method.[2] As the inhibitors are only partially soluble in water, they were dissolved in DMSO for concentrated mother solutions and DMSO/buffer for diluted solutions with a final DMSO concentration under 2,5% in all vials. Previously, the stability of the enzyme in presence of various concentrations of DMSO was controlled and the enzyme activity was unaffected.

General information for the synthesis of cyclopeptoid scaffolds 2–4

All the peptoids were synthesized in solid phase using the submonomeric approach. Solid phase syntheses were undertaken manually in polypropylene syringes fitted with two polyethylene filter discs. All solvents and soluble reagents were removed by suction. Washings between coupling and displacement steps were carried out with DMF (3 × 1 min) and DCM (3 × 1 min) using 4 mL solvent/g resin for each wash. The coupling efficiency was followed by chloranil colorimetric test. Each treatment was carried out in orbital shakers. HPLC analysis were performed on a JASCO LC-NET II/ADC instrument comprising a JASCO Model PU-2089 Plus Pump and a JASCO MD-2010 Plus UV–vis multiple wavelength detector set at 220 nm. The columns used were C_{18} reversed-phase analytical and semipreparative columns (Waters, Bondapak, 10 μ m, 125 Å, 3.9 mm × 300 mm and 7.8 × 300 mm, respectively) run with linear gradients of ACN (0.1% TFA) into H_2O (0.1% TFA) over 30 min, at a flow rate of 1.0 mL/min for the analytical runs and 2.0 mL/min for the semipreparative ones. High resolution ESI-MS spectra were recorded on a Q-Star Applied Biosystem mass spectrometer. ESI-MS analysis in positive ion mode was performed using a Finnigan LCQ Deca ion trap mass spectrometer (ThermoFinnigan, San Josè, CA, USA) and the mass spectra were acquired and processed using the Xcalibur software provided by Thermo Finnigan. The

---

[1] Behr, J.-B.; Chevrier, C.; Defoin, A.; Tarnus, C.; Streith, J. *Tetrahedron* **2003**, *59*, 543–553.
[2] Segel, I. H. *Enzyme Kinetics*; John Wiley & Sons, New York, 1975, p. 109–144.

NMR spectra were recorded on a Bruker DRX 400 (^1H at 400.13 MHz, ^{13}C at 100.03 MHz). Chemical shifts (δ) are reported in ppm relative to the residual solvent peak ($CHCl_3$, δ = 7.26; $^{13}CDCl_3$, δ = 77.0; CD_2HSOCD_3, δ = 2.50; $(^{13}CD_3)_2SO$, δ = 39.5) and the multiplicity of each signal is designated by the following abbreviations: s, singlet; d, doublet; t, triplet; m, multiplet; bs, broad singlet. Coupling constants (*J*) are quoted in hertz. The purity of all products described in the present paper has been determined by HPLC-PDA analysis at 220 nm. Yields refer to chromatographically and spectroscopically (^1H and ^{13}C NMR) pure materials.

General procedure for sub-monomer solid-phase synthesis of the linear peptoids 6–8

Linear peptoid oligomers **6**–**8** were synthesized using a submonomer solid-phase approach. In a typical synthesis 2-chlorotrityl chloride resin (α-dichlorobenzhydryl-polystyrene cross-linked with 1% DVB; 100–200 mesh; 1.5 mmol g^{-1}, 0.30 g, 0.45 mmol) was swelled in dry CH_2Cl_2 (3 mL) for 45 min and washed twice with dry DMF (3.0 mL). The first sub-monomer was attached onto the resin by adding bromoacetic acid (100 mg, 0.72 mmol) in dry CH_2Cl_2 (3.0 mL) and DIPEA (392 µL, 2.20 mmol) on a shaker platform for 60 min at room temperature, followed by washing with dry CH_2Cl_2 (3 × 1 min) and then with DMF (3 × 1 min). A DMF solution of the commercially available propargylamine (1.0 M, 3.0 mL) was added to the bromoacetylated resin. The mixture was left on a shaker platform for 30 min at room temperature, then the resin was washed with DMF (3 × 1 min), CH_2Cl_2 (3 × 1 min) and then again with DMF (3 × 1 min). Subsequent bromoacetylation reactions were accomplished by reacting the aminated oligomer with a solution of bromoacetic acid (625 mg, 4.50 mmol) and DIC (766 µL, 5.00 mmol) in dry DMF (3.0 mL) for 40 min at room temperature. The filtrated resin was washed with DMF (3 × 1 min), CH_2Cl_2 (3 × 1 min), DMF (3 × 1 min) and treated again with the amine under the same conditions reported above. This cycle of reactions was iterated until the desired chain length was accomplished and the target oligomer obtained. The cleavage was performed by treating twice the resin, previously washed with CH_2Cl_2 (3 × 1 min), with a solution of HFIP in CH_2Cl_2 (20% v/v, 6.0 mL) on a shaker platform at room temperature for 30 min and 5 min, respectively. The resin was then filtered away and the combined filtrates were concentrated in vacuo. The final products were dissolved in 50% acetonitrile in HPLC grade water and analyzed by RP-HPLC [purity >95% for oligomer **6**, 82% for oligomer **7** and 72% for oligomer **8**; conditions: 5 → 100% A in

30 min for all oligomers (A, 0.1% TFA in acetonitrile, B, 0.1% TFA in water); flow: 1.0 mL min^{-1}, 220 nm]. The linear oligomers were subjected to the cyclization reaction without further purification.

6: 70% yield (crude residue); ESI-MS: 589.2 $[M + H]^+$; t_R : 7.0 min;

7: 80% yield (crude residue); ESI-MS: 779.0 $[M + H]^+$; t_R : 7.7 min;

8: 66% yield (crude residue); ESI-MS: 969.4 $[M + H]^+$; t_R: 10.1 min;

General procedure for the cyclization reactions: synthesis of compounds 2–4

A solution of the linear peptoids **6**–**8** (0.30 mmol), previously co-evaporated three times with toluene, was prepared under nitrogen in dry DMF (20.0 mL). The solution was added dropwise to a stirred solution of HATU (465 mg, 1.22 mmol) and DIPEA (330 µL, 1.90 mmol) in dry DMF (82.0 mL) by a syringe pump over 12 h, at room temperature in anhydrous atmosphere. After 65 h the resulting mixture was concentrated in vacuo, diluted with CH_2Cl_2 (100 mL) and washed with a solution of HCl (1.0 M, 50.0 mL). The mixture was extracted with CH_2Cl_2 (2 × 100.0 mL) and the combined organic phases were washed with water (150.0 mL), dried over anhydrous $MgSO_4$, filtered and concentrated in vacuo. The cyclic products were dissolved in 50% acetonitrile in HPLC grade water and analyzed by RP-HPLC [purity >95% for oligomers **2** and **3**, 81% for oligomer **4**, conditions: 5–100% A in 30 min for all the cycles (A, 0.1% TFA in acetonitrile, B, 0.1% TFA in water); flow: 1 mL min^{-1}, 220 nm]. The crude residue **2** was purified by precipitation in hot acetonitrile, **3** by precipitation in hot acetonitrile with 0.1% TFA, while **4** was purified by RP-HPLC on a C18 reversed-phase semi-preparative column, t_R : 10.9 min, conditions: 25–100% A in 30 min [(A, 0.1% TFA in acetonitrile, B, 0.1% TFA in water); flow: 2 mL min^{-1}, 220 nm].

Compound **2**: 31%; white amorphous solid; ESI-MS: 593.2 m/z $[M + Na]^+$, t_R: 8.6 min;

^{1}H NMR: (400 MHz, DMSO, mixture of rotamers) δ : 5.60–2.90 (m, 30 H, $COCH_2N$, NCH_2CCH, NCH_2CCH, water- overlapped signals).

^{13}C NMR: (100 MHz, DMSO, mixture of rotamers, broad signals) δ: 170.7, 169.8, 169.5, 169.3, 169.2, 169.0, 168.9, 168.8, 168.6, 168.4, 168.2, 167.9 , 167.8, 167.7, 167.1, 79.5, 79.4, 79.3, 79.1, 79.0, 78.8, 78.7, 78.5, 78.4, 78.3, 78.2, 78.0, 77.9, 76.3, 76.2, 76.1, 75.9, 75.7, 75.6, 75.4, 75.2, 75.1, 74.7, 74.5, 49.4, 49.3, 49.0, 48.4, 48.3, 47.9, 47.7, 47.6, 47.4, 47.3, 47.0, 46.8, 46.7, 37.2, 36.8, 36.6, 36.4, 36.2, 36.0, 35.7, 35.3, 35.1, 34.7, 34.5, 34.4.

Compound **3**: 32%; white amorphous solid; ESI-MS: 761.3 m/z $[M + H]^+$, 783.3 m/z $[M + Na]^+$; t_R: 9.7 min;

^{1}H NMR: (400 MHz, $CDCl_3$, mixture of rotamers) δ: 4.97 (m, 4 H, COCH_2N,), 4.75 (d, J= 16.8 Hz, 1H, COCH_2N), 4.61 – 3.83 (m, 23 H, COCH_2N and NCH_2CCH), 3.71 (m, 3 H, COCH_2N), 3.34 (d, J= 16.8 Hz 1H, COCH_2N,), 2.46 (t, J= 2.1 Hz, 2 H, NCH_2CC*H*), 2.39 (t, J= 2.3 Hz, 2 H, NCH_2CC*H*), 2.32 (t, J= 2.5 Hz, 2 H, NCH_2CC*H*), 2.22 (t, J= 2.4 Hz, 2 H, NCH_2CC*H*).

^{13}C NMR: (100 MHz, $CDCl_3$, mixture of rotamers) δ : 169.0, 168.4, 168.2, 167.0, 78.3, 78.1, 77.2, 76.7, 74.6, 74.2, 73.6, 73.3, 49.4, 48.6, 46.7, 46.5, 38.6, 38.1, 36.5, 35.8.

Compound **4**: 12%, by HPLC; pale yellow amorphous solid; ESI-MS: 951.3 m/z $[M + H]^+$, 973.3 m/z $[M + Na]^+$; t_R : 10.9 min;

^{1}H-NMR: (400 MHz, $CDCl_3$, mixture of rotamers) δ: 4.74 – 3.77 (m, 37 H, COCH_2N and NCH_2CCH), 3.33 (bs, 3 H, NCH_2CCH), 2.49 – 2.00 (m, 10 H, NCH_2CC*H*).

^{13}C-NMR: (100 MHz, $CDCl_3$, mixture of rotamers, broad signals) δ: 169.6 , 169.4, 169.1, 168.7, 168.5, 168.2, 168.1, 167.9, 167.7, 167.4, 167.1, 78.2, 78.0, 77.8, 77.6, 74.8, 74.6, 74.4, 74.2, 74.0, 73.8, 73.7, 73.5, 73.3, 73.2, 73.1, 72.8, 49.0, 48.8, 48.5, 48.2, 47.9, 47.7, 47.5, 47.1, 46.7, 46.4, 46.2, 38.7, 38.6, 38.3, 38.1, 38.0, 37.8, 37.6, 37.4, 37.2, 37.0, 36.8, 36.6, 36.4, 36.2, 36.0, 35.8, 35.6.

^{1}H-NMR spectrum (DMSO, 400 MHz) for compound 2:

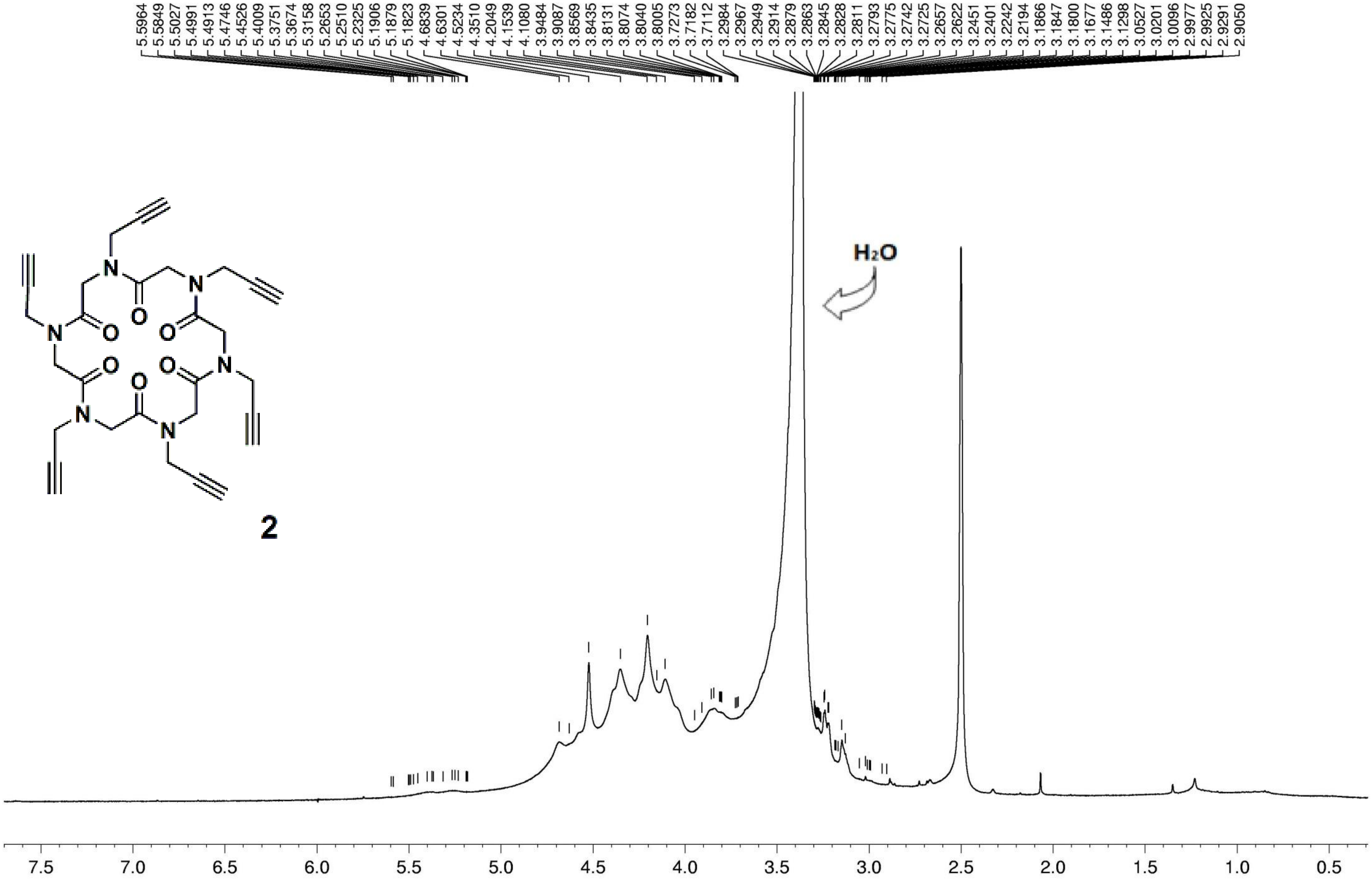

2

^{13}C-NMR spectrum (DMSO, 100 MHz) for compound 2:

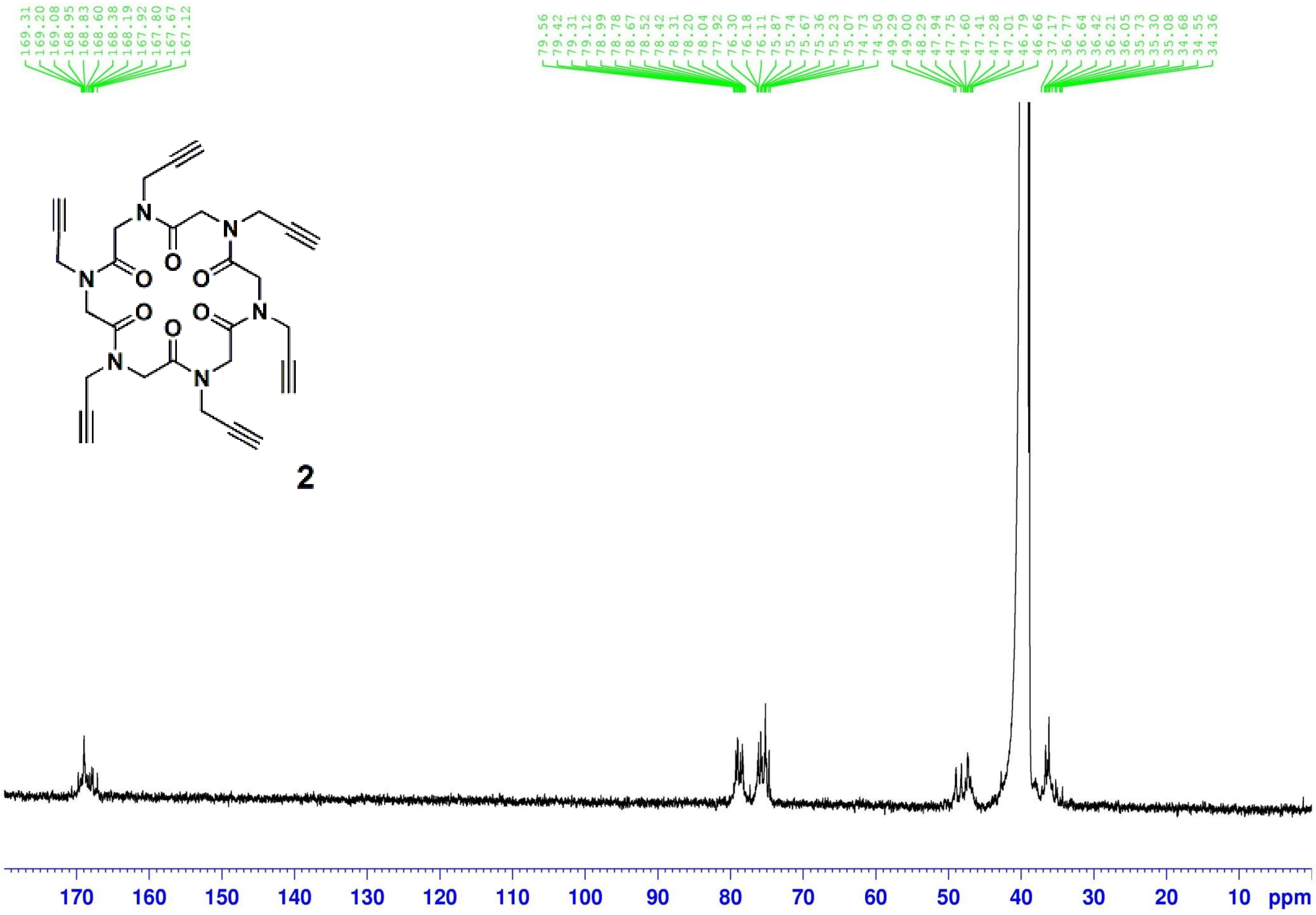

^{1}H-NMR spectrum ($CDCl_3$, 400 MHz) for compound 3:

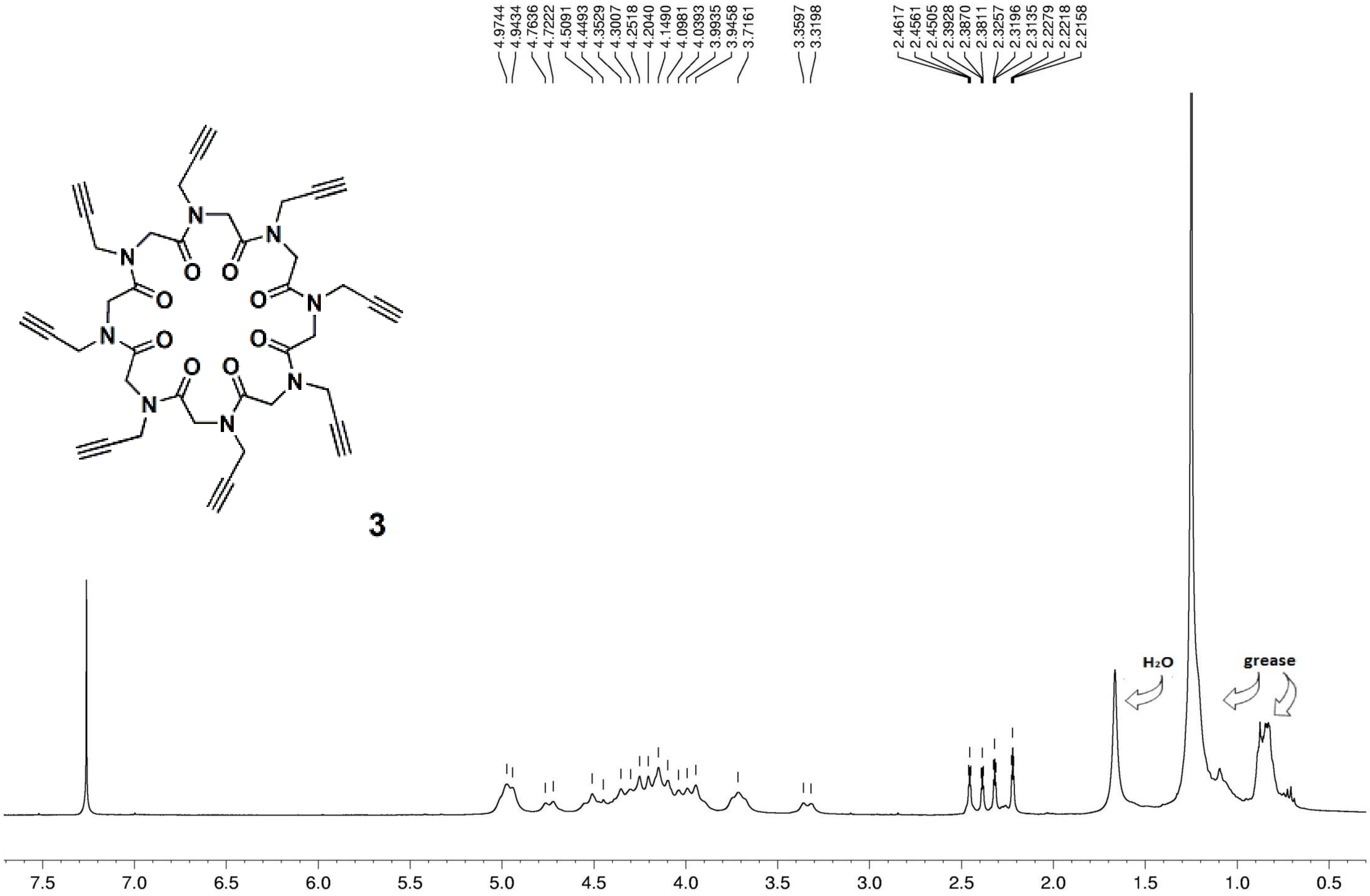

^{13}C-NMR spectrum ($CDCl_3$, 100 MHz) for compound 3:

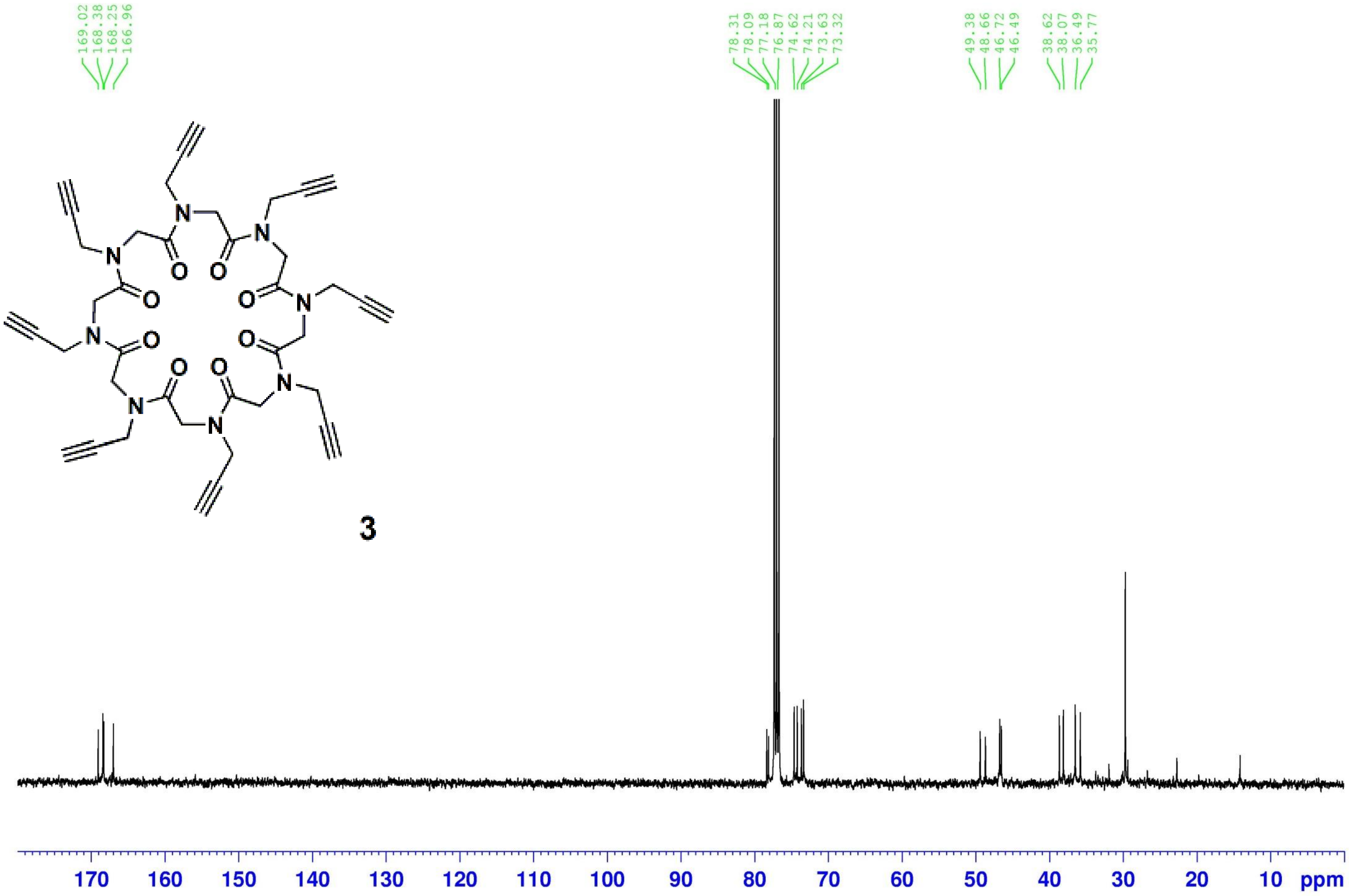

^{1}H NMR spectrum ($CDCl_3$, 400 MHz) for compound 4:

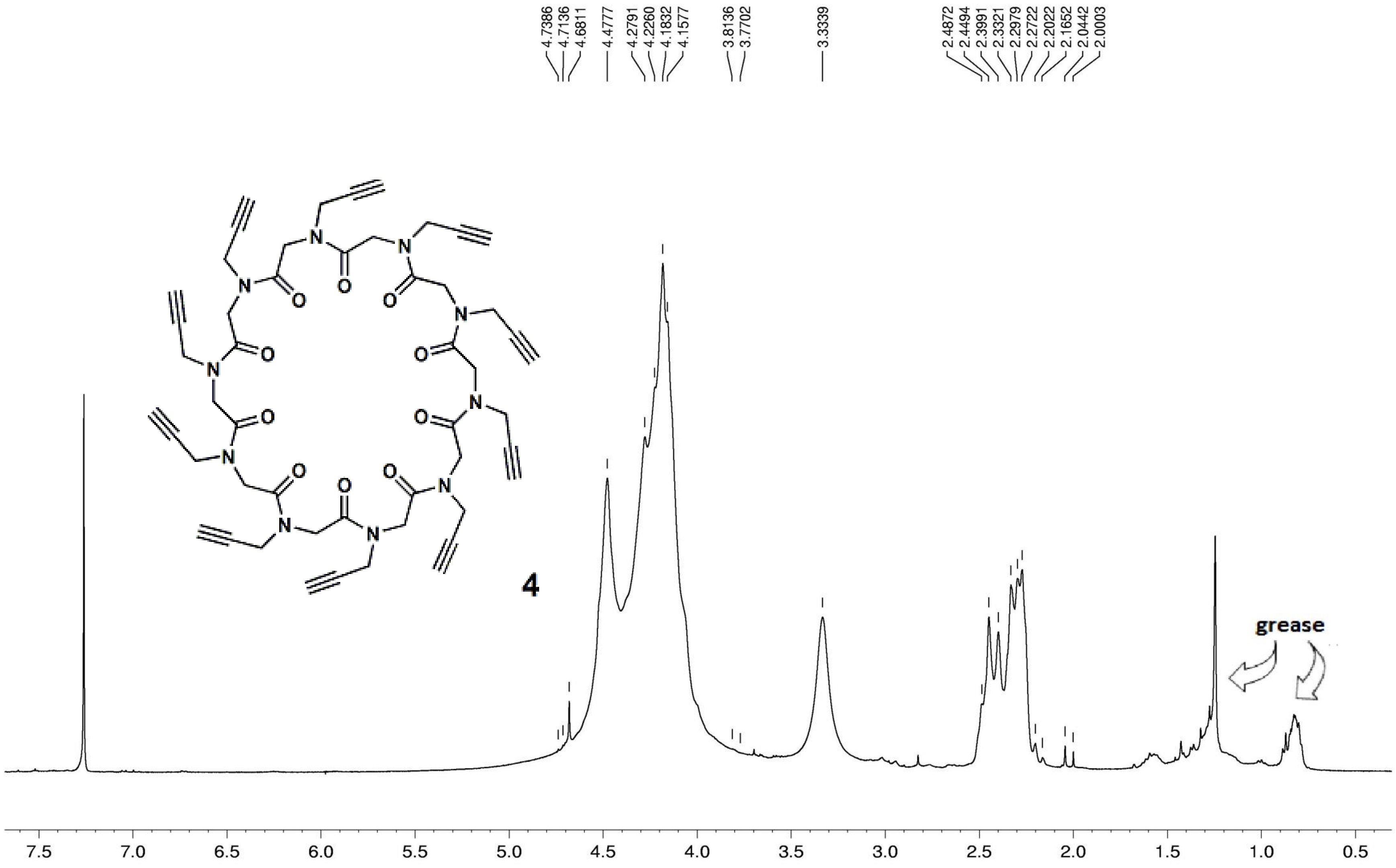

^{13}C-NMR spectrum ($CDCl_3$, 100 MHz) for compound 4:

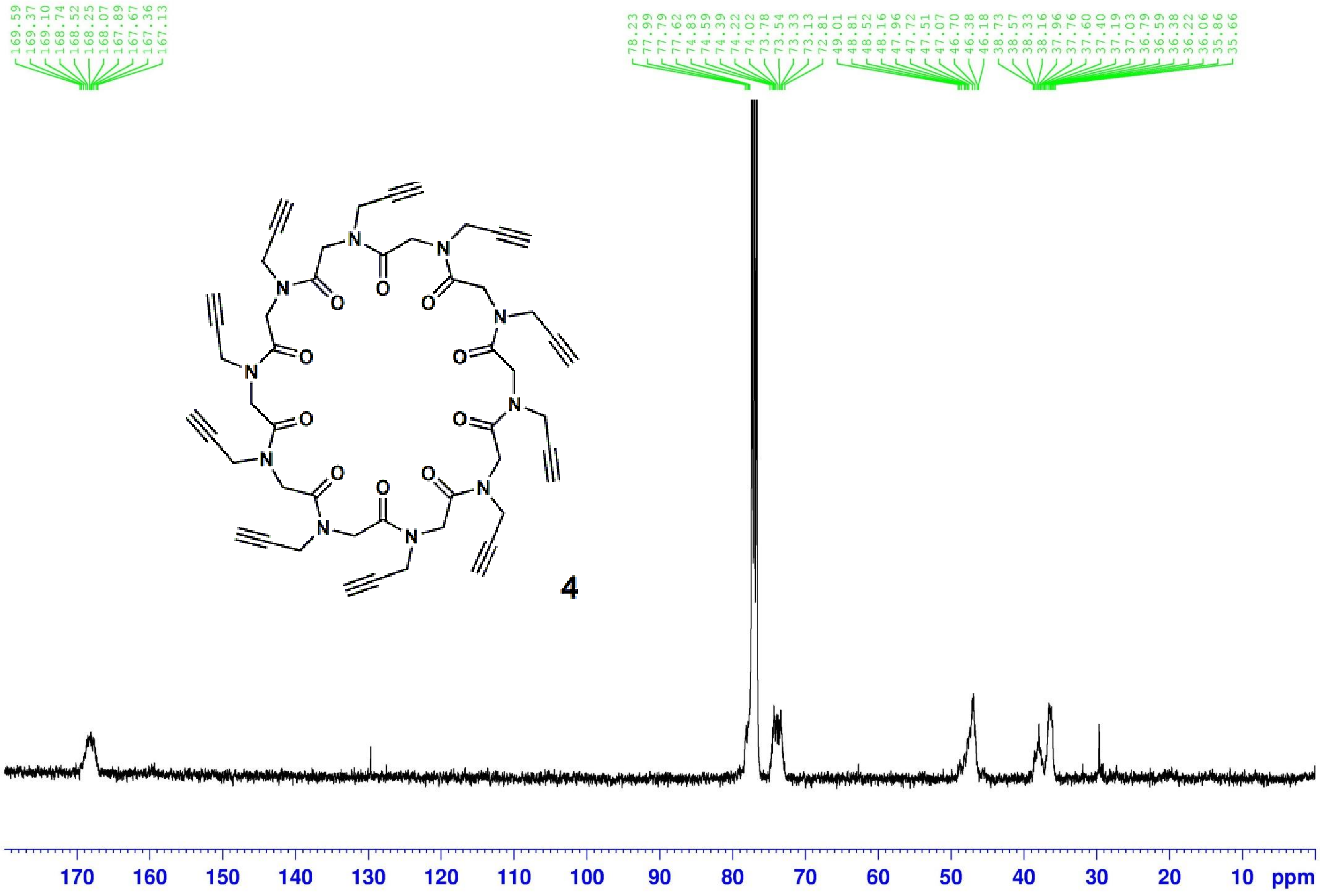

4

Analytical data and copies of the ^{1}H NMR and ^{13}C NMR spectra of DNJ clusters 9–10

Compound 9b:

$[\alpha]_{D}^{20} = +5.8$ (c 1, $CHCl_3$); ^{1}H-NMR ($CD_3CN/CDCl_3$ 9:1 + 9 eq. sodium picrate, 400 MHz): δ 7.76 (s, 6H; H-1'), 4.97 (m, J = 10.3 Hz, 12H; H-3, H-4), 4.85 (d, J = 16.3 Hz, 6H; H-3' or H-5'), 4.83 (td, J = 9.8, 5.3 Hz, 6H; H-2), 4.71 (d, J = 16.3 Hz, 6H; H-3' or H-5'), 4.45 (d, J = 16.3 Hz, 6H; H-3' or H-5'), 4.32 (t, J = 7.0 Hz, 12H; H-15), 4.10 (dd, J = 19.4, 13.0 Hz, 12H; H-6), 3.97 (d, J = 16.3 Hz, 6H; H-3' or H-5'), 3.11 (dd, J = 11.1, 5.3 Hz, 6H; H-1a), 2.70 (m, 6H; H-7a), 2.68 (d, J = 8.8 Hz, 6H; H-5), 2.51 (m, 6H; H-7b), 2.35 (dd, J = 12.7, 11.1 Hz, 6H; H-1b), 1.95 (s, 72H; AcO), 1.85 (m, 12H; H-14), 1.40 (m, 12H; H-8), 1.28 ppm (m, 60H; H-9 to H-13); ^{13}C-NMR ($CD_3CN/CDCl_3$ 9:1, 100 MHz): δ 171.4, 170.94, 170.88, 170.6, 170.6-168.9, 144.4-142.8, 124.7-123.8, 75.3, 70.5, 70.2, 62.1, 60.5, 53.4, 52.3, 50.8, 50.3-48.5, 44.9-42.6, 30.9, 30.2, 30.1, 29.7, 27.9, 27.2, 25.3, 21.1 ppm; HRMS (ESI): m/z calcd for $C_{168}H_{261}N_{30}O_{54}$ $[M + 3H]^{3+}$ 1187.6194; found 1187.6215.

Compound 9c:

$[\alpha]_{D}^{24} = +5.1$ (c 1, $CHCl_3$); ^{1}H-NMR ($CD_3CN/CDCl_3$ 9:1, 400 MHz): δ 7.78-76.8 (m, 8H; H-1’), 5.26-5.10 (m, 4H; H-3’ or H-5’), 4.98 (m, 16H; H-3 and H-4), 4.95-3.83 (m, 28H; H-3’ and H-5’), 4.84 (m, 8H; H-2), 4.33 (m, 16H; H-12), 4.12 (s, 16H; H-6), 3.11 (m, 8H; H-1a), 2.69 (m, 16H; H-5 and H-7a), 2.55 (m, 8H; H-7b), 2.37 (m, 8H; H-1b), 1.99 (s, 96H; AcO), 1.88 (m, 16H; H-11), 1.42 (br s, 16H; H-8), 1.31 ppm (br s, 32H; H-9 and H-10); ^{13}C-NMR ($CD_3CN/CDCl_3$ 9:1, 100 MHz): δ 171.2, 170.83, 170.79, 170.7, 170.5, 169.8, 169.7, 167.5, 144.1, 143.2, 124.2, 124.1, 124.0, 123.8, 75.2, 70.3, 70.1, 61.9, 60.4, 53.3, 52.2, 50.7, 50.0, 49.2, 48.8, 47.9, 44.4, 43.5, 42.55, 30.7, 27.1, 26.9, 25.1, 21.0 ppm; HRMS (ESI): m/z calcd for $C_{200}H_{299}N_{40}O_{72}$ $[M + 3H]^{3+}$ 1471.0316; found 1471.0329.

Compound 9d:

$[\alpha]_{D}^{21} = +5.9$ (c 1, $CHCl_3$); ^{1}H-NMR ($CD_3CN/CDCl_3$ 9:1, 400 MHz): δ 7.78-76.8 (m, 8H; H-1’), 5.26-5.10 (m, 4H; H-3’ or H-5’), 4.99 (m, 16H; H-3 and H-4), 4.95-3.83 (m, 28H; H-3’ and H-5’), 4.85 (td, J = 9.7, 5.1 Hz, 8H; H-2), 4.33 (m, 16H; H-15), 4.13 (s, 16H; H-6), 3.14 (dd, J = 11.7, 4.8 Hz, 8H; H-1a), 2.69 (m, 16H; H-5 and H-7a), 2.55 (m, 8H; H-7b), 2.37 (dd, J = 11.9, 10.3 Hz, 8H; H-1b), 1.99 (s, 96H; AcO), 1.88 (m, 16H; H-14), 1.42 (br s, 16H; H-8), 1.31 ppm (br s, 80H; H-9 to H-13); ^{13}C-NMR ($CD_3CN/CDCl_3$ 9:1, 100 MHz): δ 171.2, 170.83, 170.76, 170.7, 170.5, 169.8, 169.7, 167.5, 144.1, 143.14, 143.07, 124.17, 124.05, 123.94, 123.8, 75.2, 70.3, 70.1, 61.9, 60.4, 53.3, 52.2, 50.7, 50.0, 49.2, 48.8, 47.9, 44.4, 43.5, 42.55, 30.8, 30.0, 29.6, 27.7, 27.1, 25.3, 21.0 ppm; HRMS (ESI): m/z calcd for $C_{224}H_{345}N_{40}O_{72}Na$ $[M + H + Na]^{2+}$ 2385.2226; found 2385.2264.

Compound 9e:

$[\alpha]_{D}^{20} = +4.5$ (c 1, $CHCl_3$); ^{1}H-NMR ($CD_3CN/CDCl_3$ 9:1, 400 MHz): δ 8.05-7.64 (m, 10H; H-1’), 5.58-3.48 (br m, 40H; H-3’ and H-5’), 4.98 (m, 20H; H-3 and H-4), 4.84 (m, 10H; H-2), 4.34 (br s, 20H; H-12), 4.12 (m, 20H; H-6), 3.12 (br s, 10H; H-1a), 2.69 (m, 20H; H-5 and H-7a), 2.56 (m, 10H; H-7b), 2.37 (m, 10H; H-1b), 1.99 (s, 120H; AcO), 1.87 (m, 20H; H-11), 1.51-1.15 ppm

(m, 60H; H-8 to H-10); ^{13}C-NMR ($CD_3CN/CDCl_3$ 9:1, 75.5 MHz): δ 171.9, 171.4, 171.3, 171.1, 171.0-169.2, 145.0-143.3, 125.6-124.1, 75.7, 70.9, 70.7, 62.5, 61.0, 53.9, 52.8, 51.4, 50.4-48.9, 45.2-42.3, 31.4, 27.8, 27.5, 25.8, 21.6 ppm; HRMS (ESI): m/z calcd for $C_{250}H_{372}N_{50}O_{90}$ [M + 2H]$^{2+}$ 2757.3029; found 2757.3300.

Compound 9f:
$[\alpha]_D^{21} =$ +6.5 (*c* 1, $CHCl_3$); ^{1}H-NMR ($CDCl_3$, 400 MHz): δ 8.14-7.44 (m, 10H; H-1'), 5.86-3.30 (br m, 40H; H-3' and H-5'), 5.04 (m, 20H; H-3 and H-4), 4.93 (m, 10H; H-2), 4.33 (br s, 20H; H-15), 4.14 (s, 20H; H-6), 3.18 (m, 10H; H-1a), 2.71 (m, 10H; H-7a), 2.62 (d, J = 8.8Hz, 10H; H-5), 2.54 (m, 10H; H-7b), 2.31 (dd, J = 11.6, 9.9 Hz, 10H; H-1b), 2.04 (m, 120H; AcO), 1.89 (m, 20H; H-14), 1.51-1.05 ppm (m, 120H; H-8 to H-13); ^{13}C-NMR ($CD_3CN/CDCl_3$ 9:1, 100 MHz): δ 171.4, 170.9, 170.8, 170.6, 170.2-168.9, 144.4-142.2, 125.0-123.5, 75.3, 70.4, 70.2, 62.0, 60.5, 53.4, 52.5, 50.9, 50.4-47.0, 45.3-41.5, 31.0, 30.2, 30.1, 29.7, 27.9, 27.2, 25.4, 21.1 ppm; MS (MALDI-TOF): m/z calcd for average mass of $C_{280}H_{430}N_{50}O_{90}Na$ [M + Na]$^+$ 5959.1; found 5958.7.

Compound 10b:
$[\alpha]_D^{20} =$ -14.0 (*c* 0.0625, H_2O/DMSO 1:1 + 0.1% TFA); ^{1}H-NMR (D_2O/CD_3OD 1:1, 400 MHz): δ 8.13-7.68 (m, 6H; H-1'), 5.09-3.89 (br m, 12H; H-3' and H-5'), 4.36 (s, 12H; H-15), 3.80 (dd, J = 19.6, 12.8 Hz, 12H; H-6), 3.47 (td, J = 9.2, 5.0 Hz, 6H; H-2), 3.32 (t, J = 9.6 Hz, 6H; H-4), 3.16 (t, J = 9.4 Hz, 6H; H-3), 2.93 (dd, J = 10.6, 5.0 Hz, 6H; H-1a), 2.67 (s, 6H; H-7a), 2.56 (s, 6H; H-7b), 2.20 (dd, J = 10.6, 9.2 Hz, 6H; H-1b), 2.13 (d, J = 9.6 Hz, 6H; H-5), 1.83 (s, 12H; H-14), 1.40 (s, 12H; H-8), 1.23 ppm (s, 60H; H-9 to H-13); ^{13}C-NMR (D_2O + 0.1% TFA, 125 MHz): δ 173.5-169.4, 144.6-142.7, 127.1-124.7, 77.5, 68.7, 67.8, 66.7, 55.2, 54.8, 54.7, 52.2, 51.7-48.8, 46.4-43.2, 31.0, 30.2-28.9, 27.7-26.4, 24.8 ppm; HRMS (ESI): m/z calcd for $C_{120}H_{212}N_{30}O_{30}$ [M + 2H]$^{2+}$ 1276.7987; found 1276.7991.

Compound 10c:
$[\alpha]_D^{20} =$ -9.6 (*c* 1, H_2O); ^{1}H-NMR (D_2O/CD_3OD 1:1, 400 MHz): δ 8.05-7.77 (m, 8H; H-1'), 5.11-4.02 (br m, 32H; H-3' and H-5'), 4.37 (m, 16H; H-12), 3.77 (m, 16H; H-6), 3.47 (br s, 8H; H-2), 3.31 (m, 8H; H-4), 3.16 (m, 8H; H-3), 2.91 (br s, 8H; H-1a), 2.66 (br s, 8H; H-7a), 2.53 (br s, 8H, H-7b), 2.14 (m, 16H; H-1b and H-5), 1.85 (br s, 16H; H-11), 1.39 (br s, 16H; H-8), 1.25 ppm (m, 32H; H-9 and H-10); ^{13}C-NMR (D_2O/CD_3OD 1:1, 100 MHz): δ 171.7, 171.4, 170.9, 170.3, 169.3, 144.2, 144.1, 143.5, 143.3, 125.5, 79.8, 71.4, 70.9, 70.2, 66.5, 59.1, 56.9, 53.3, 51.5, 50.9-49.8, 44.7-43.2, 30.7, 27.6, 26.9, 24.0 ppm; MS (MALDI-TOF): m/z calcd for $C_{136}H_{233}N_{40}O_{40}$ [M + H]$^+$ 3066.7; found 3066.8.

Compound 10d:
$[\alpha]_D^{20} =$ -10.0 (*c* 1, MeOH); ^{1}H-NMR (CD_3OD, 400 MHz): δ 8.11-7.80 (m, 8H; H-1'), 5.25-3.99 (br m, 32H; H-3' and H-5'), 4.40 (m, 16H; H-15), 3.84 (s, 16H; H-6), 3.49 (td, J = 9.5, 4.5 Hz, 8H; H-2), 3.36 (t, J = 9.0 Hz, 8H; H-4), 3.16 (t, J = 9.0 Hz, 8H; H-3), 2.98 (dd, J = 9.8, 4.4 Hz, 8H; H-1a), 2.75 (m, 8H; H-7a), 2.57 (m, 8H; H-7b), 2.18 (t, J = 10.7 Hz, 8H; H-1b), 2.12 (d, J = 10.9 Hz, 8H; H-5), 1.89 (br s, 16H; H-14), 1.47 (br s, 16H; H-8), 1.31 ppm (m, 80H; H-9 to H-13);

^{13}C-NMR (CD_3OD, 100 MHz): δ 172.0, 171.2, 170.6, 169.3, 144.7, 144.5, 143.9, 143.6, 125.4, 125.33, 125.29, 125.23, 80.6, 72.1, 70.8, 67.3, 59.6, 57.7, 53.9, 51.6, 51.0-49.6, 44.6, 44.3, 44.0, 43.7, 31.4, 30.6, 30.2, 28.7, 27.6, 25.1 ppm; MS (MALDI-TOF): m/z calcd for $C_{160}H_{281}N_{40}O_{40}$ $[M + H]^+$ 3403.1; found 3403.3.

Compound 10e:

$[\alpha]_D^{20}$ = -10.2 (c 1, H_2O); ^{1}H-NMR (D_2O, 500 MHz): δ 8.15-7.62 (m, 10H; H-1'), 5.69-3.85 (br m, 40H; H-3' and H-5'), 4.35 (m, 20H; H-12), 3.76 (m, 20H; H-6), 3.47 (m, 10H; H-2), 3.30 (m, 10H; H-4), 3.17 (m, 10H; H-3), 2.90 (m, 10H; H-1a), 2.61 (m, 10H; H-7a), 2.50 (m, 10H; H-7b), 2.15 (m, 20H; H-1b and H-5), 1.80 (m, 20H; H-11), 1.35 (m, 20H; H-8), 1.18 ppm (m, 40H; H-9 and H-10); ^{13}C-NMR (D_2O, 125 MHz): δ 171.5-167.7, 143.5-141.1, 124.6, 78.3, 70.0, 68.8, 65.0, 57.5, 55.3, 51.9, 50.4, 49.3-47.0, 43.9-41.5, 29.4, 26.1, 25.5, 22.5 ppm; HRMS (ESI): m/z calcd for $C_{170}H_{292}N_{50}O_{50}$ $[M + 2H]^{2+}$ 1917.0916; found 1917.1349.

Compound 10f:

$[\alpha]_D^{20}$ = -6.5 (c 0.5, MeOH/H_2O 1:1); ^{1}H-NMR (D_2O/CD_3OD 1:1, 400 MHz): δ 8.16-7.71 (m, 10H; H-1'), 5.64-4.03 (br m, 40H; H-3' and H-5'), 4.37 (m, 20H; H-15), 3.82 (m, 20H; H-6), 3.50 (t, J = 9.7, 4.7 Hz, 10H; H-2), 3.35 (t, J = 9.7 Hz, 10H; H-4), 3.18 (t, J = 9.4 Hz, 10H; H-3), 2.96 (d, J = 9.4 Hz, 10H; H-1a), 2.70 (m, 10H; H-7a), 2.59 (m, 10H; H-7b), 2.22 (t, J = 11.1 Hz, 10H; H-1b), 2.16 (d, J = 9.5 Hz, 10H; H-5), 1.86 (m, 20H; H-14), 1.43 ppm (m, 20H; H-8), 1.24 (m, 100H; H-9 to H-13); ^{13}C-NMR (CD_3CN/CD_3OD 1:1, 100 MHz): δ 172.4-168.4, 144.7-141.6, 124.7, 79.6, 71.1, 69.9, 66.3, 58.7, 56.8, 53.1, 51.1, 50.3, 59.0, 44.1-42.0, 30.6, 29.9, 29.4, 28.0, 26.9, 24.0 ppm; MS (MALDI-TOF): m/z calcd for $C_{200}H_{351}N_{50}O_{50}$ $[M + H]^+$ 4253.7; found 4254.1.

^{1}H NMR spectrum ($CD_3CN/CDCl_3$ 9:1 + 11 equiv sodium picrate, 400 MHz) for compound 9a:

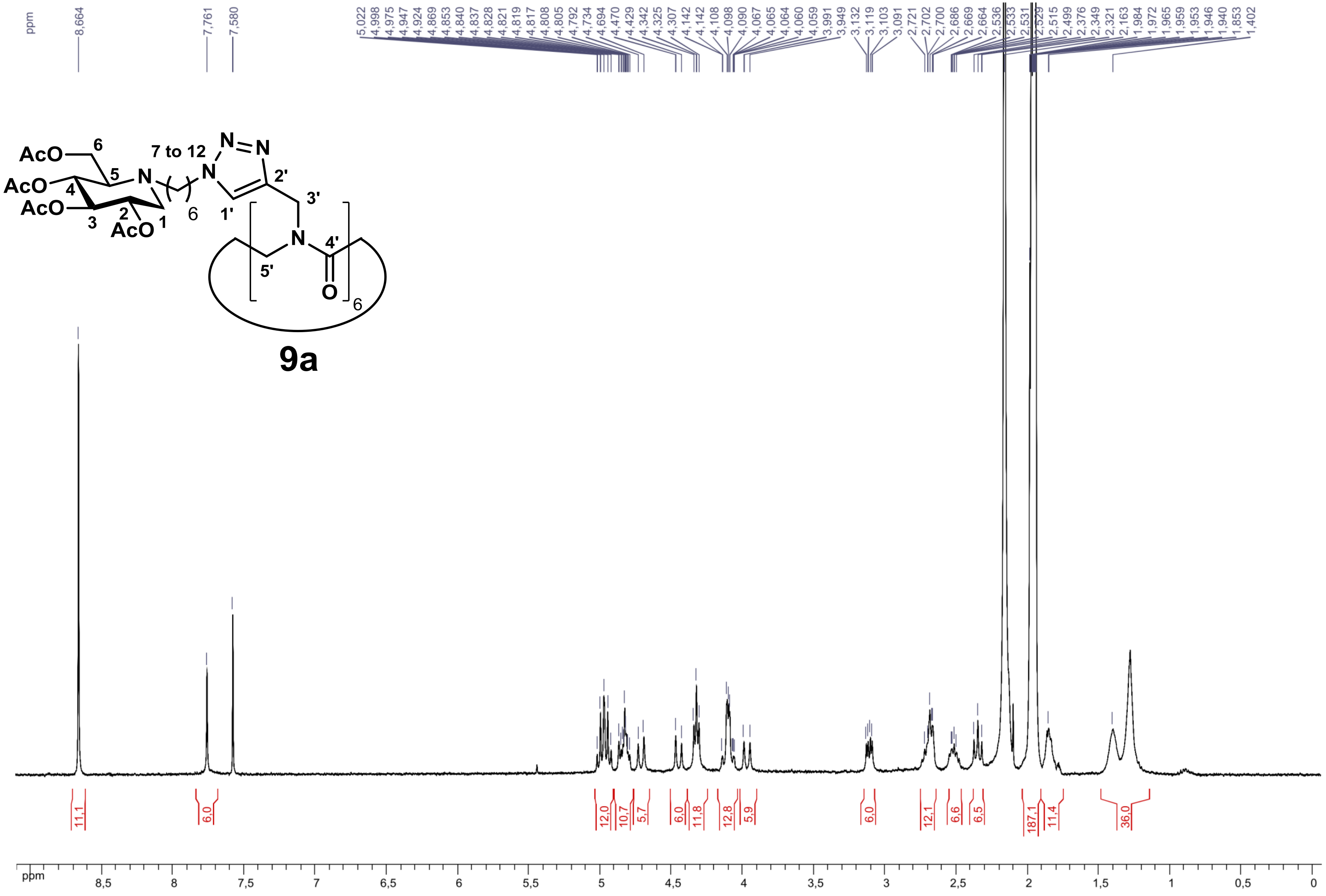

9a

^{13}C NMR spectrum ($CD_3CN/CDCl_3$ 9:1, 100 MHz) for compound 9a:

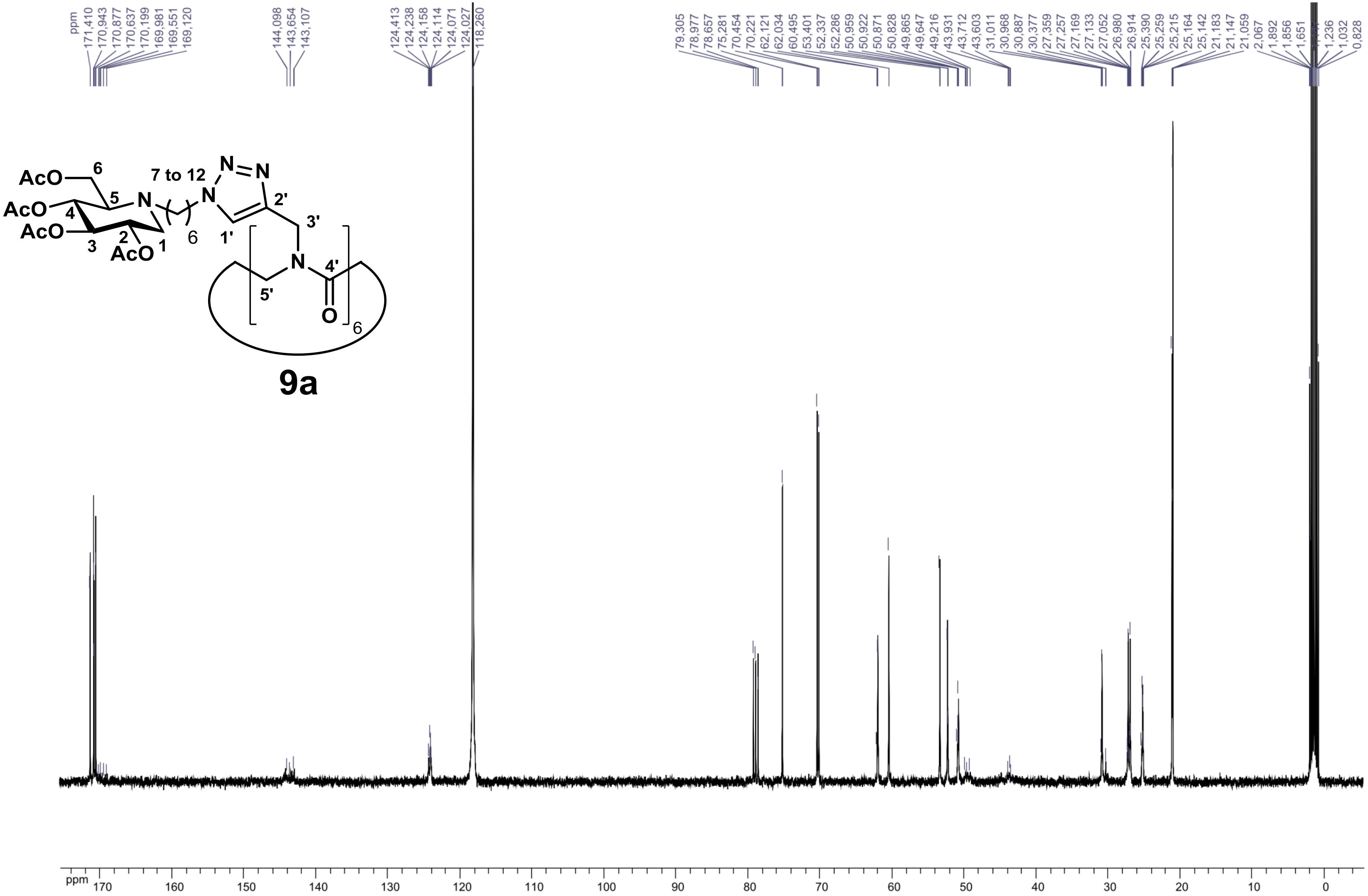

^{1}H NMR spectrum ($CD_3CN/CDCl_3$ 9:1 + 9 equiv sodium picrate, 400 MHz) for compound 9b:

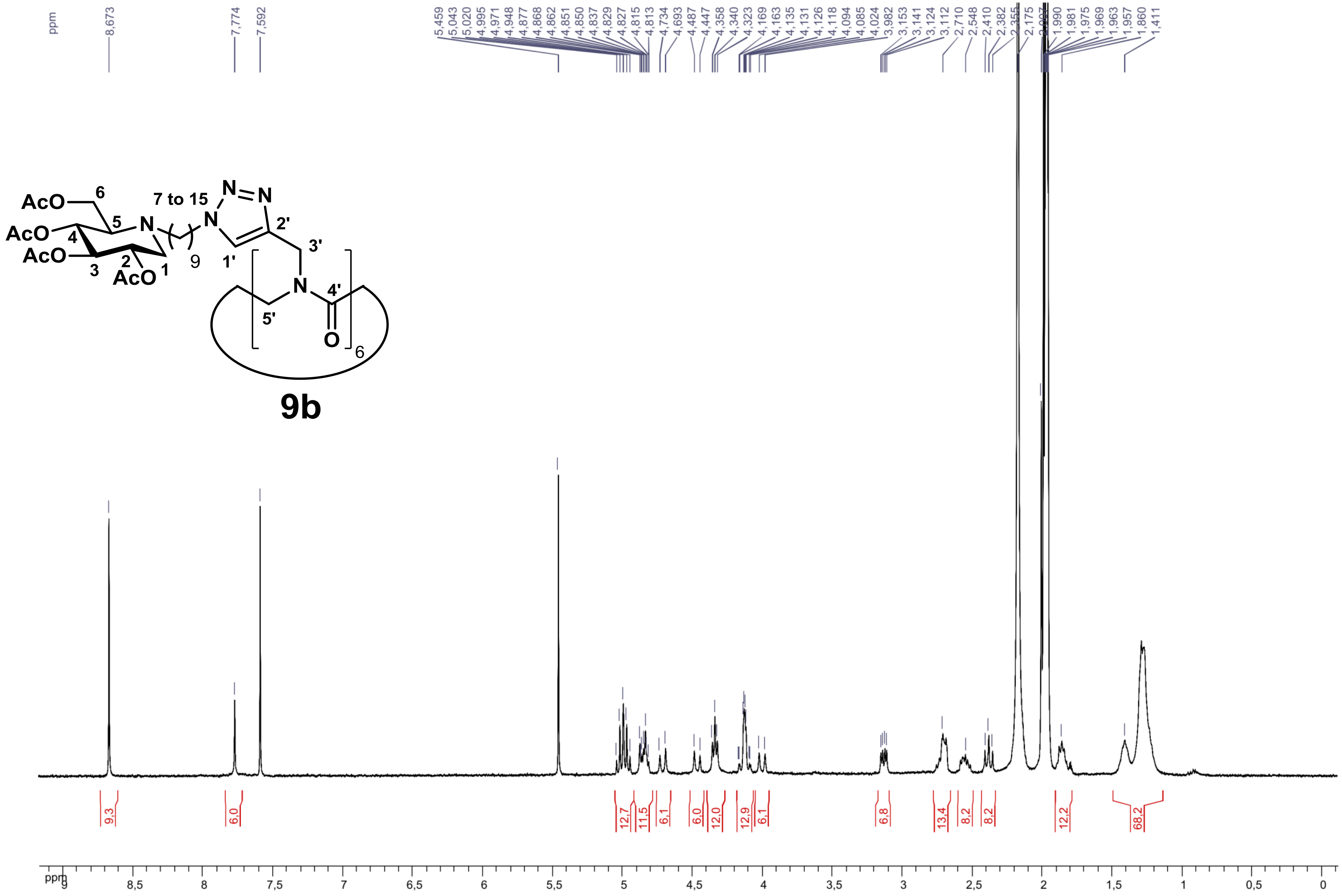

^{13}C NMR spectrum ($CD_3CN/CDCl_3$ 9:1, 100 MHz) for compound 9b:

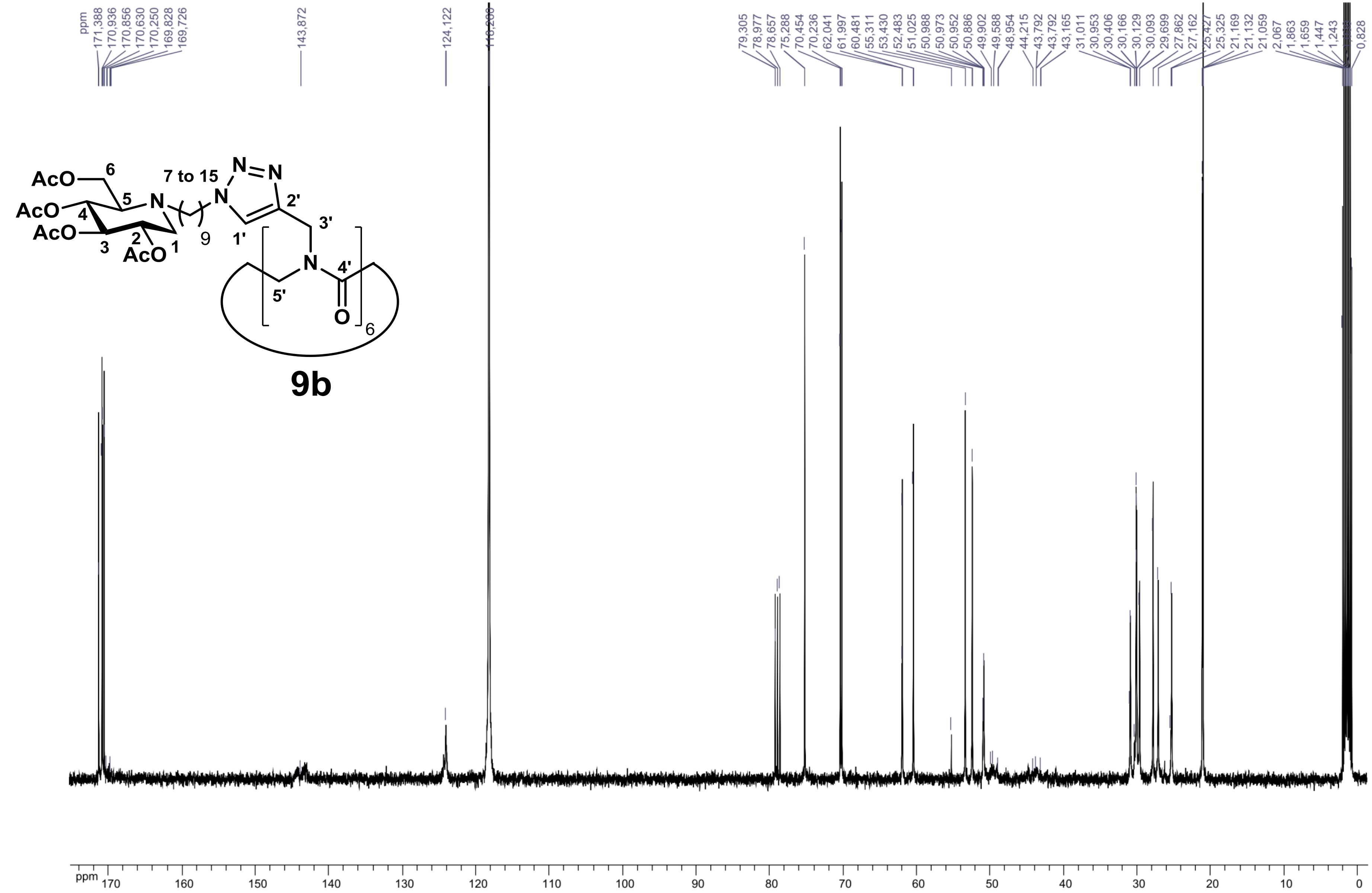

^{1}H NMR spectrum ($CD_3CN/CDCl_3$ 9:1, 400 MHz) for compound 9c:

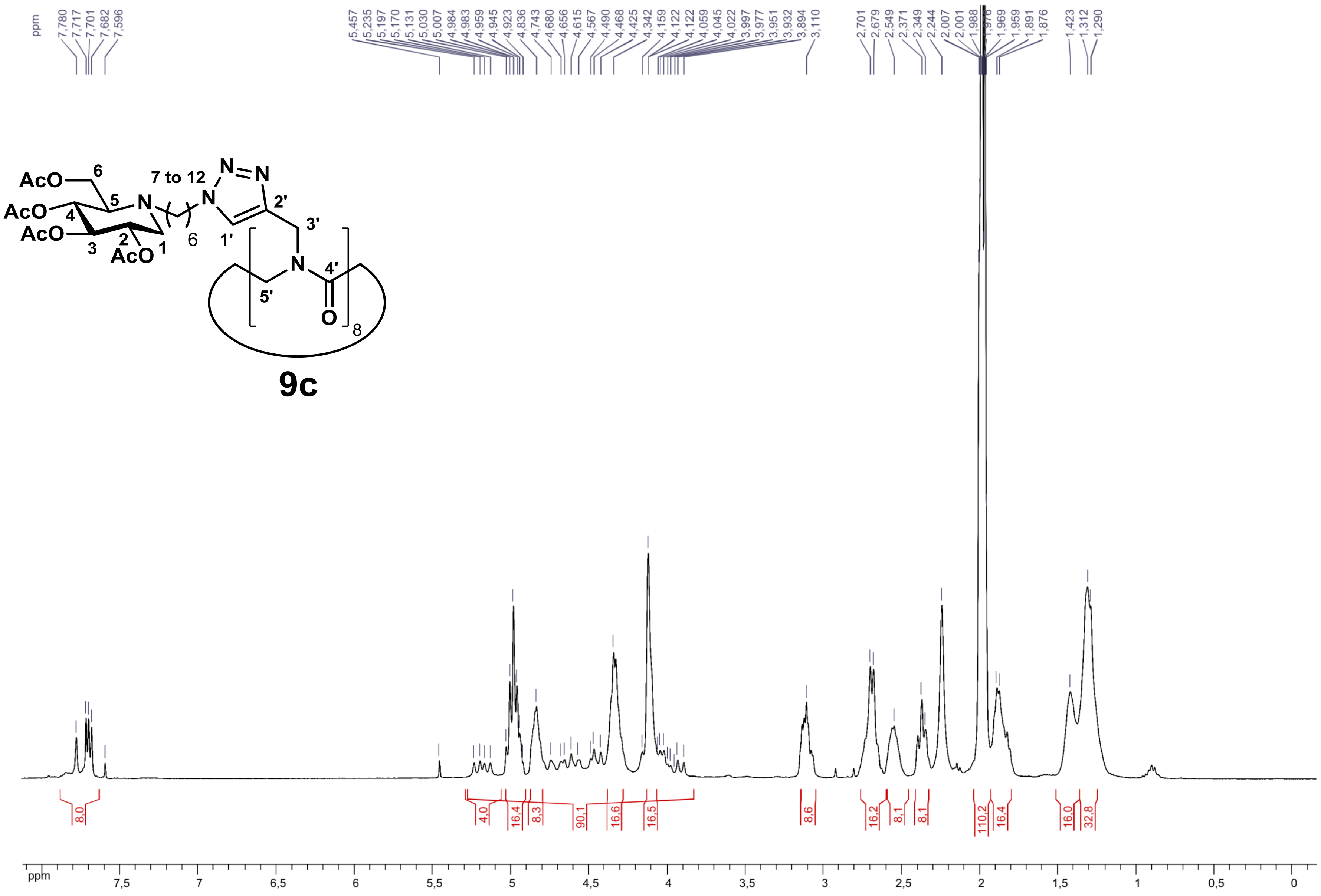

^{13}C NMR spectrum ($CD_3CN/CDCl_3$ 9:1, 100 MHz) for compound 9c:

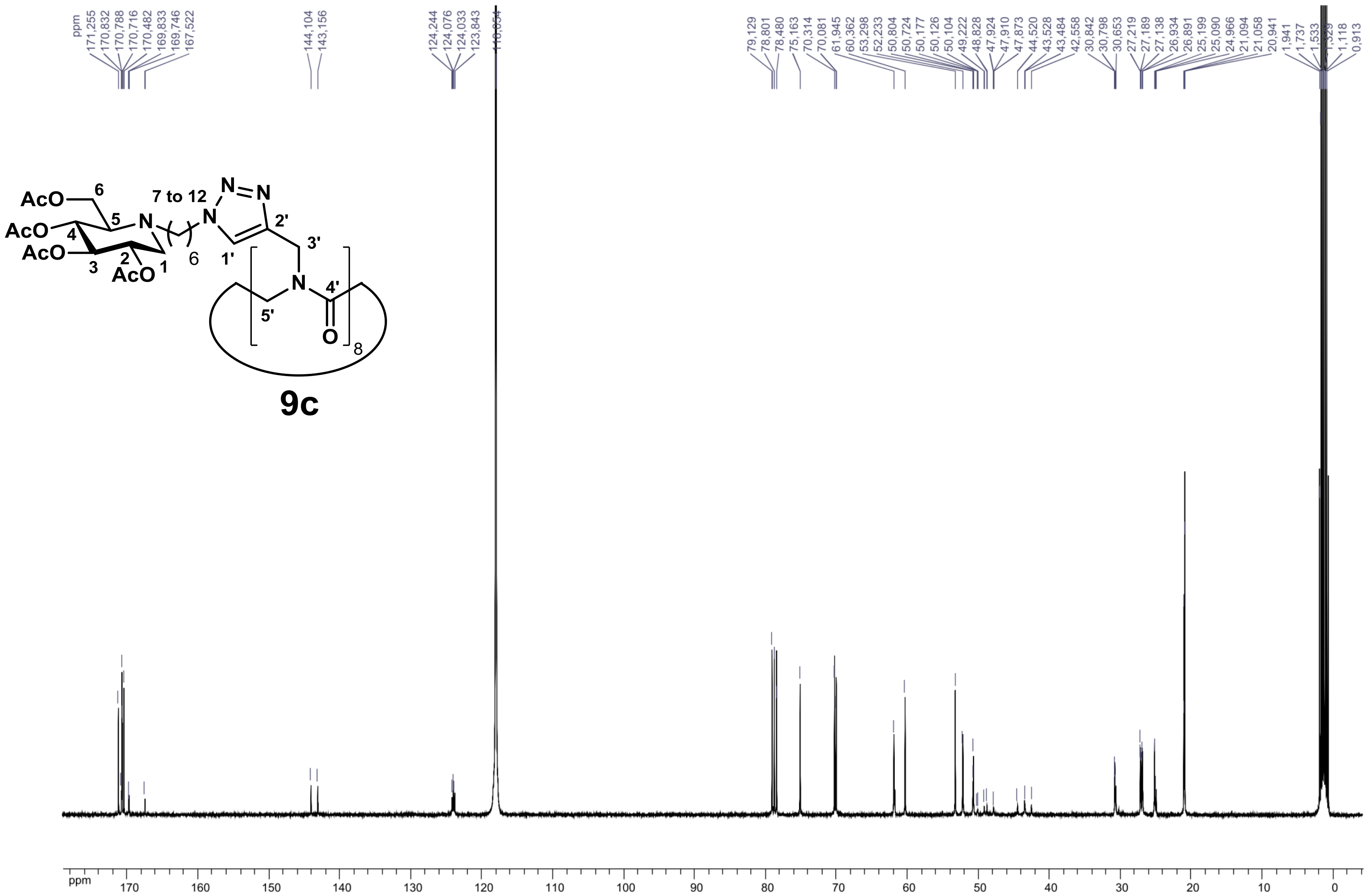

9c

^{1}H NMR spectrum ($CD_3CN/CDCl_3$ 9:1, 400 MHz) for compound 9d:

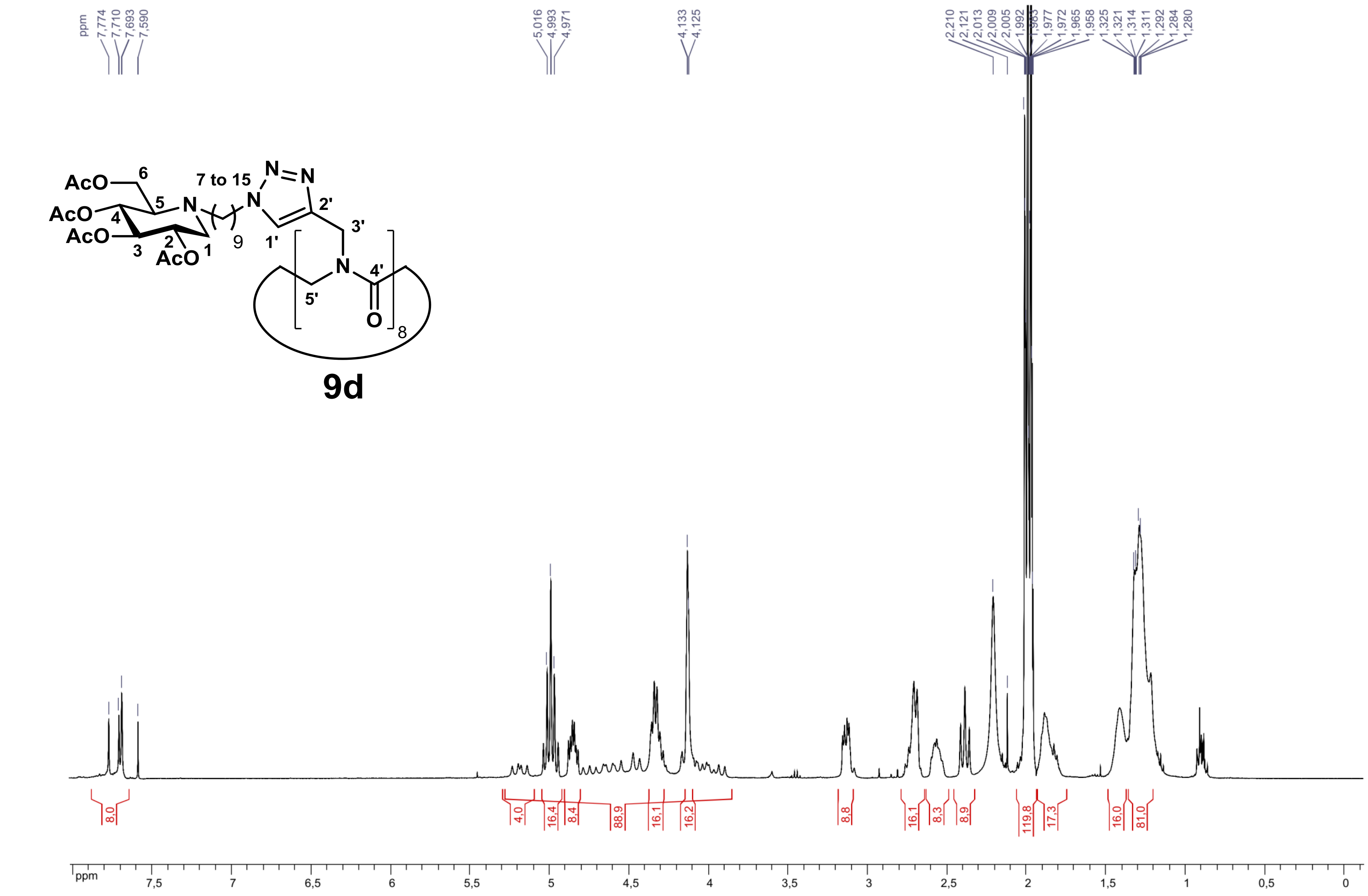

^{13}C NMR spectrum ($CD_3CN/CDCl_3$ 9:1, 100 MHz) for compound 9d:

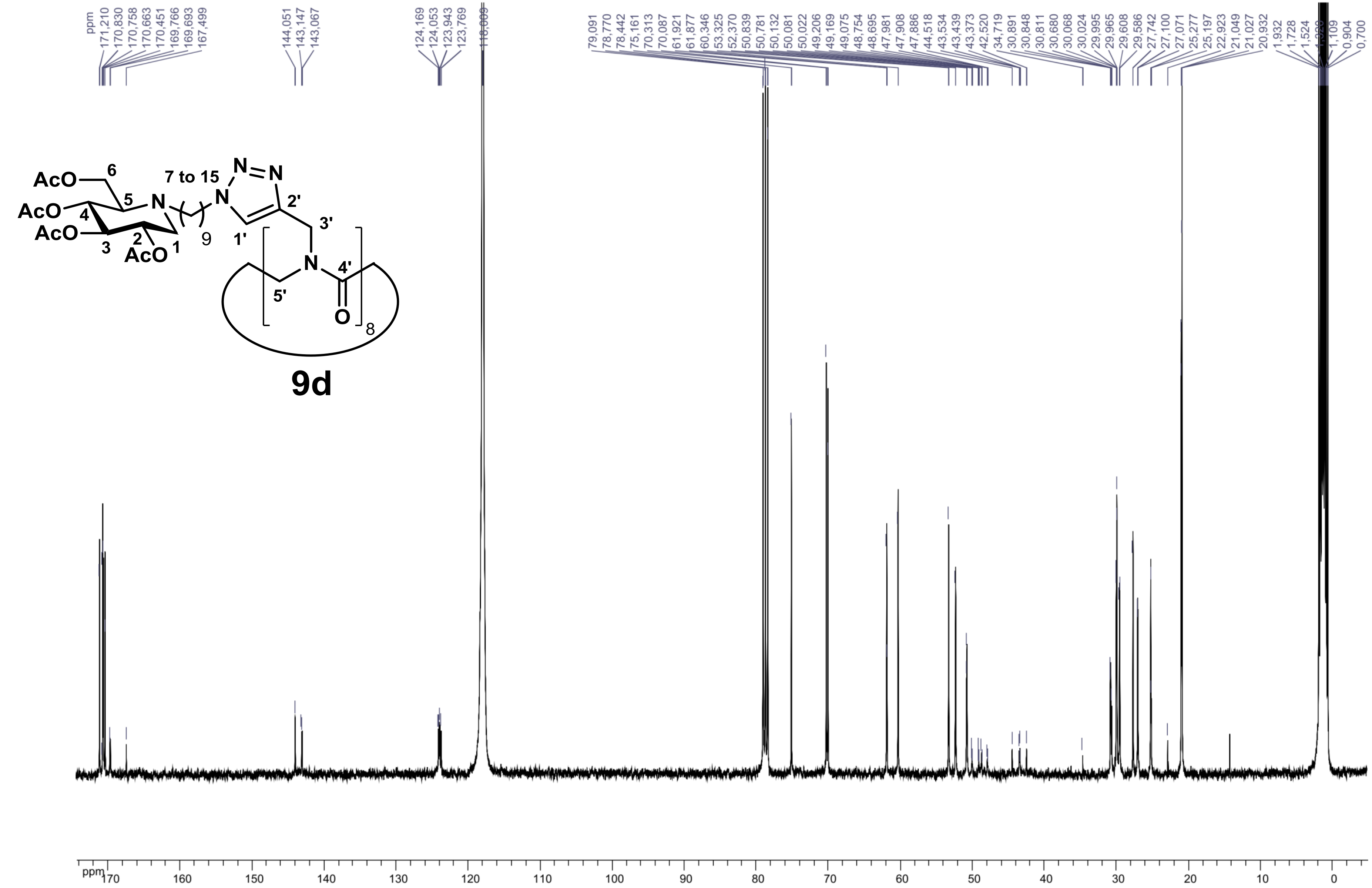

^{1}H NMR spectrum ($CD_3CN/CDCl_3$ 9:1, 400 MHz) for compound 9e:

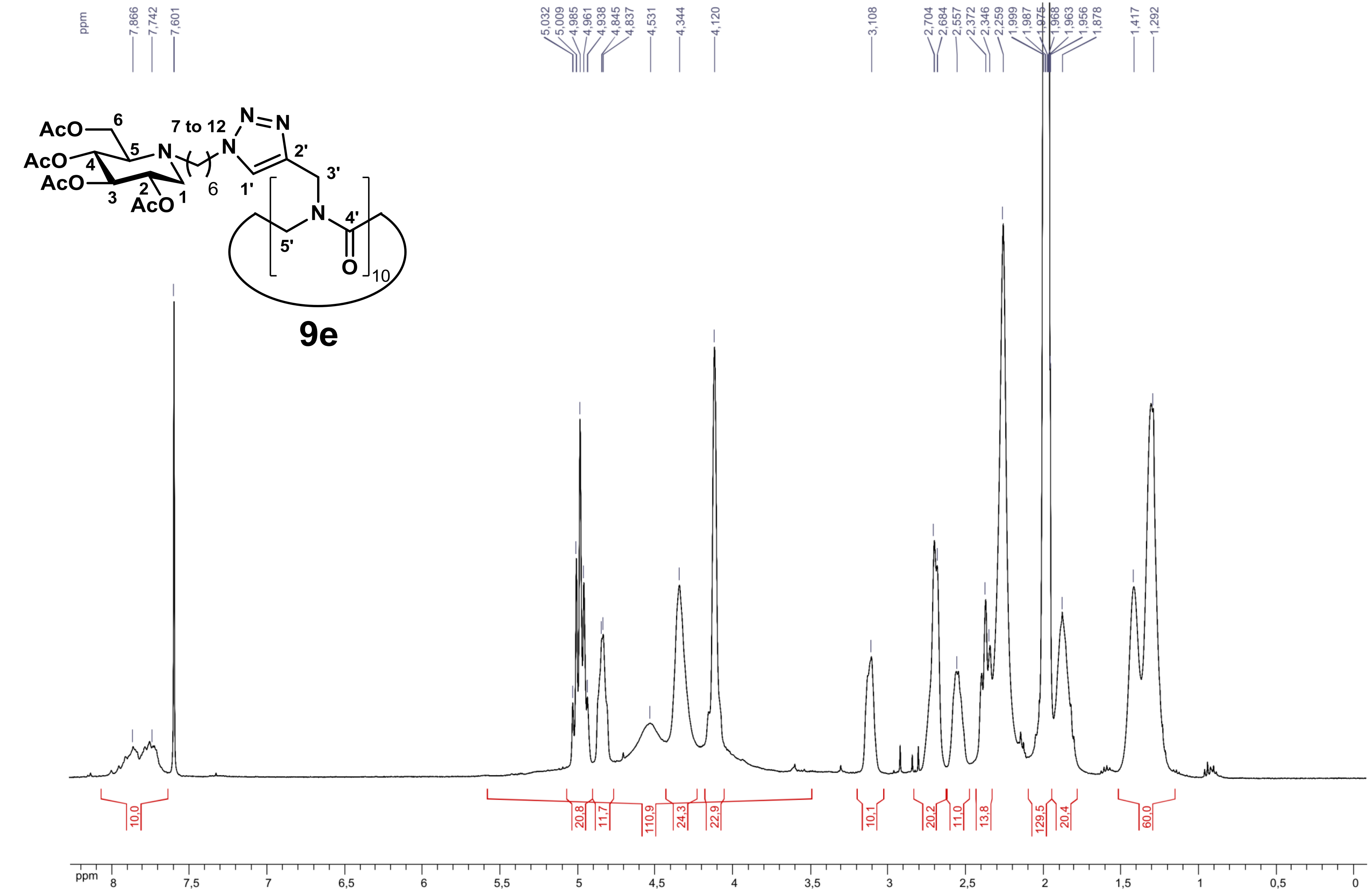

^{13}C NMR spectrum ($CD_3CN/CDCl_3$ 9:1, 75.5 MHz) for compound 9e:

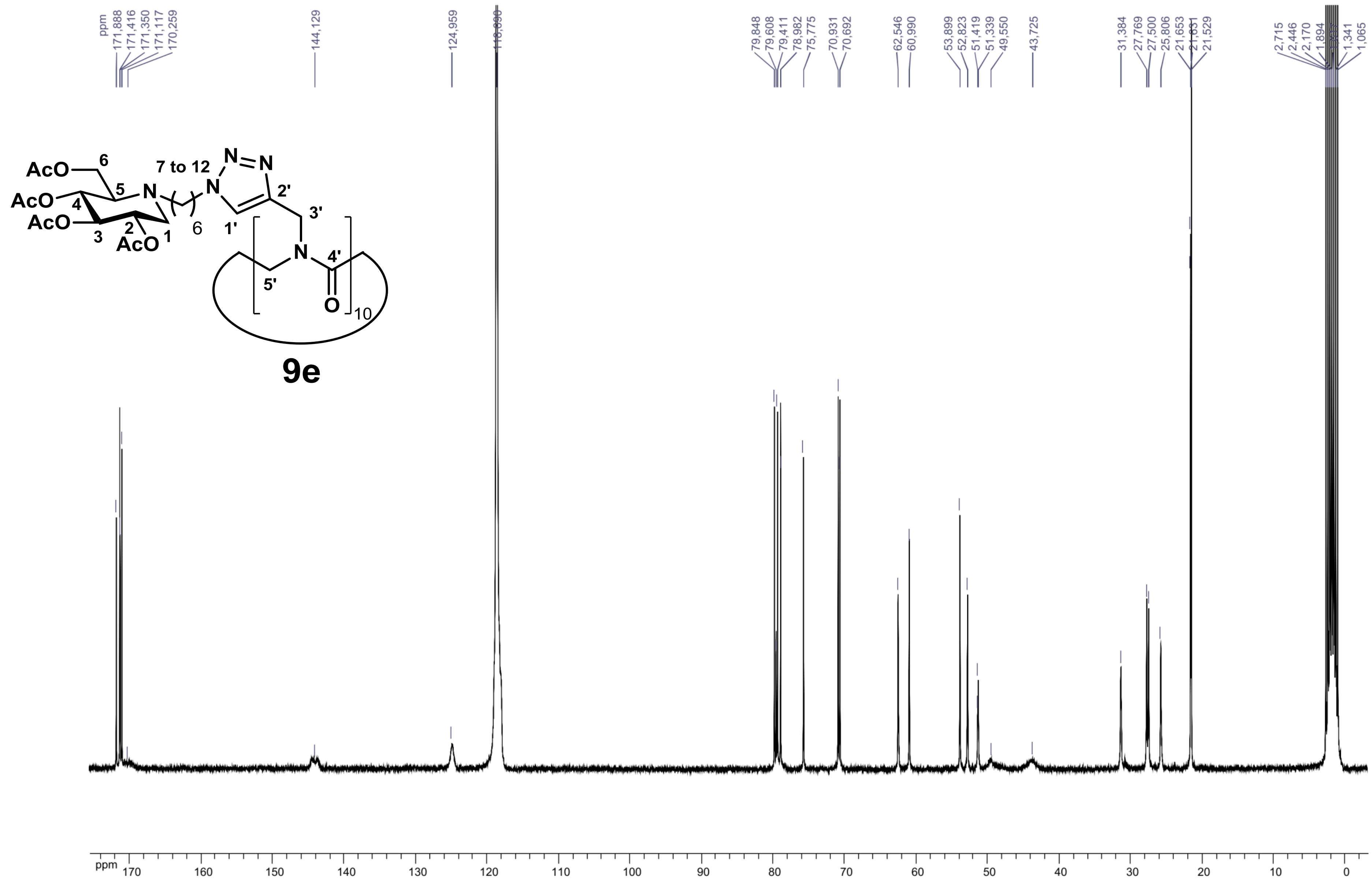

^{1}H NMR spectrum ($CDCl_3$, 400 MHz) for compound 9f:

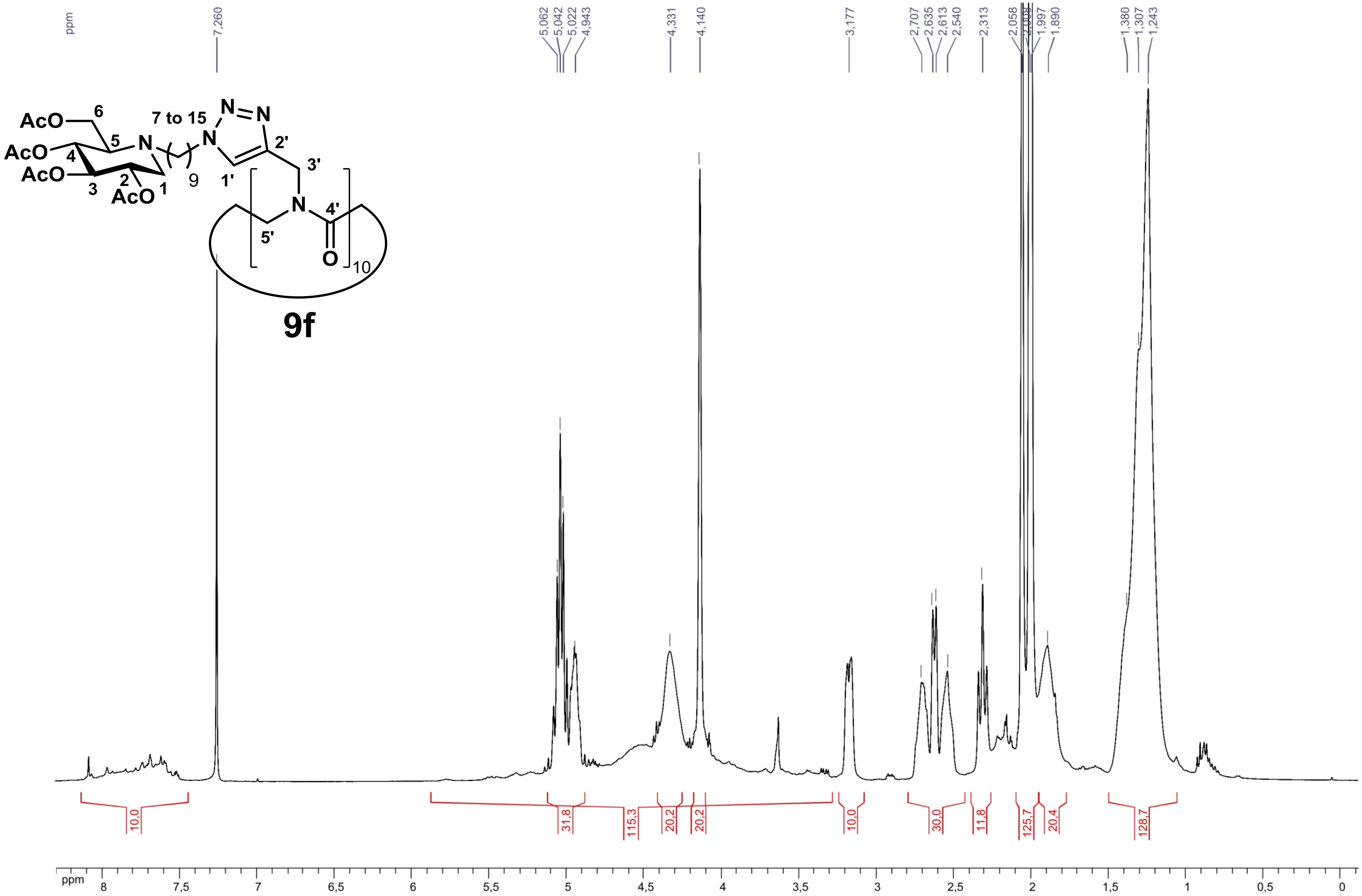

^{13}C NMR spectrum ($CD_3CN/CDCl_3$ 9:1, 100 MHz) for compound 9f:

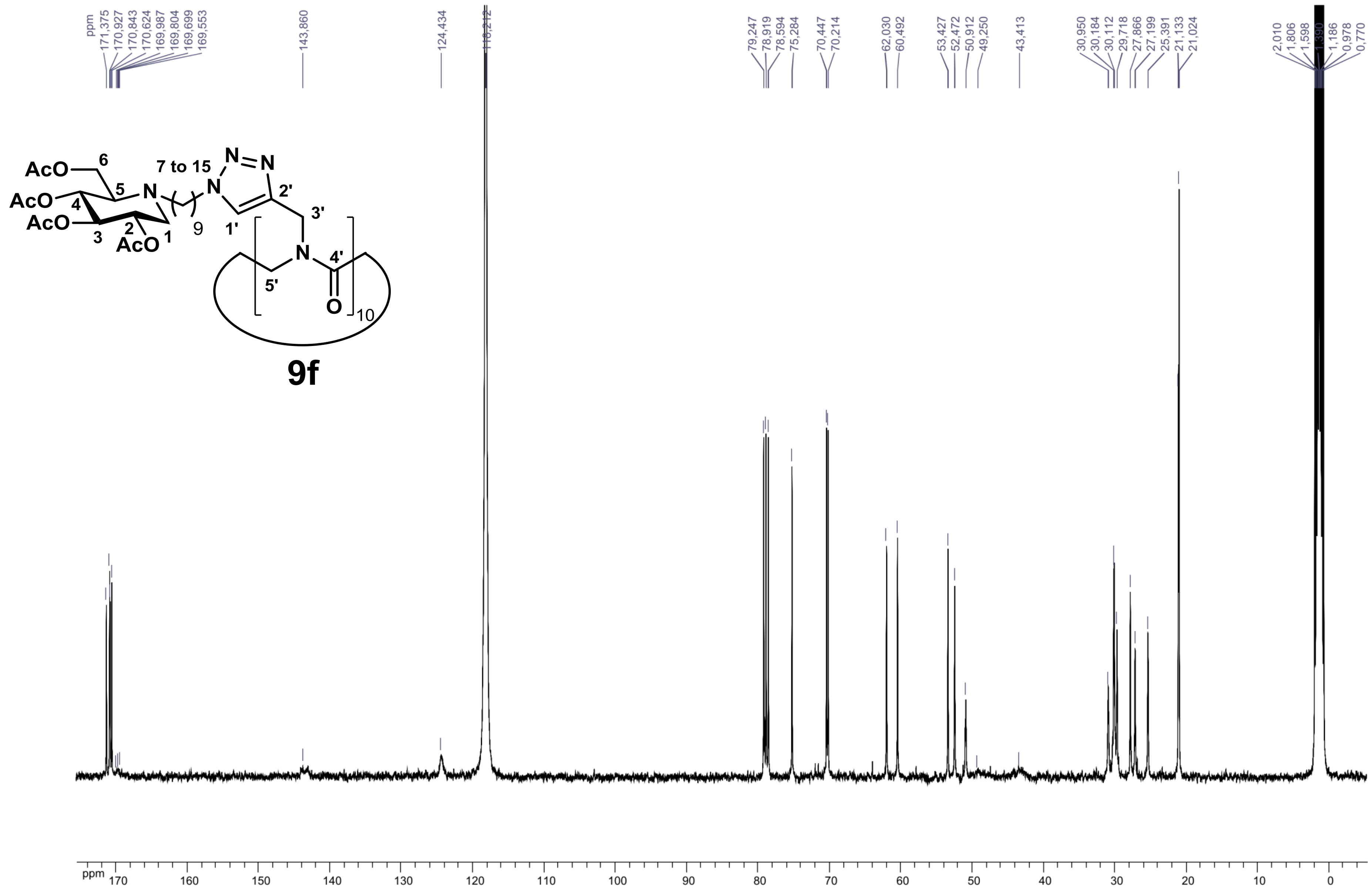

^{1}H NMR spectrum (D_2O + 0.1% TFA, 500 MHz) for compound 10a:

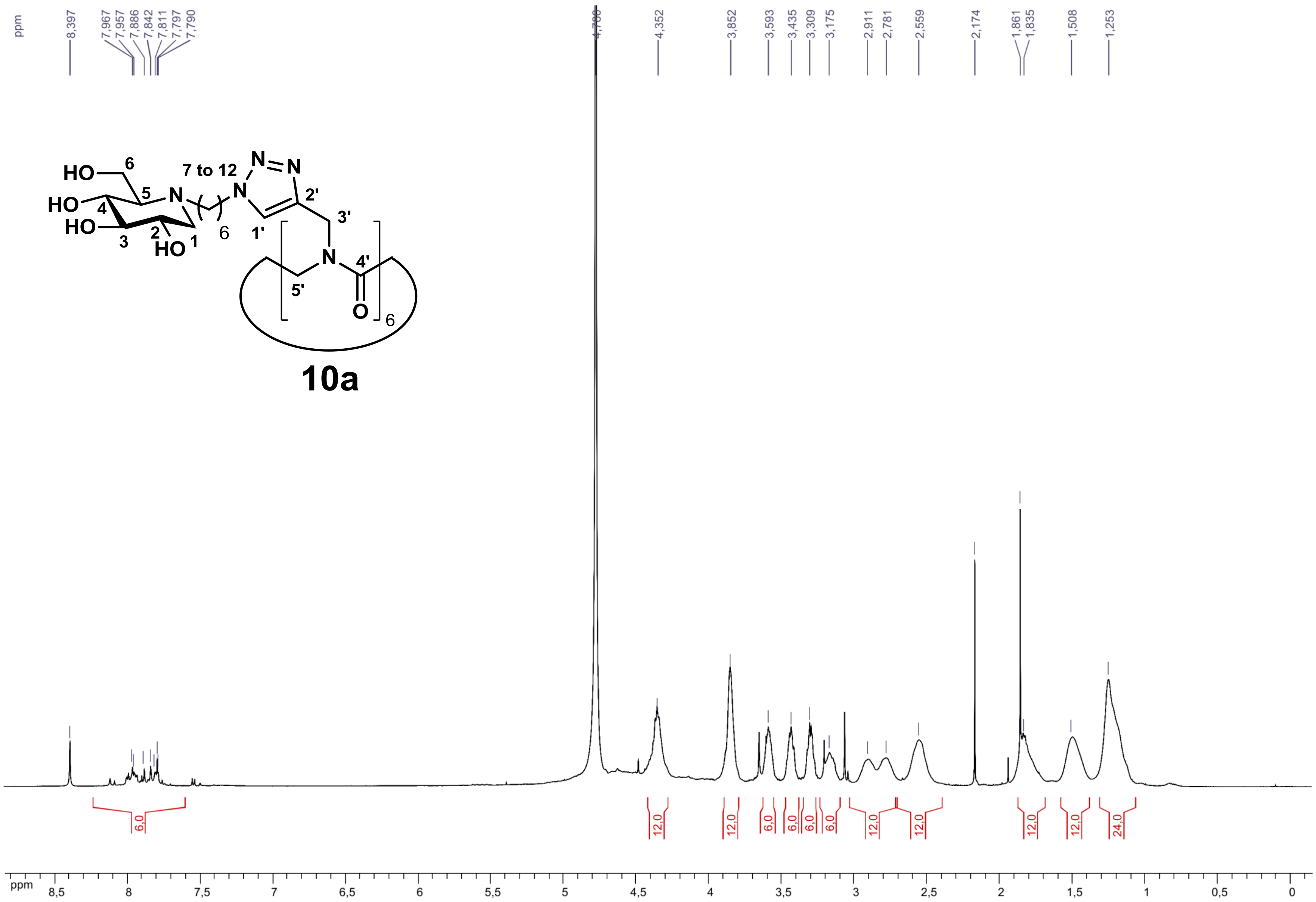

^{13}C NMR spectrum (D_2O + 0.1% TFA, 125 MHz) for compound 10a:

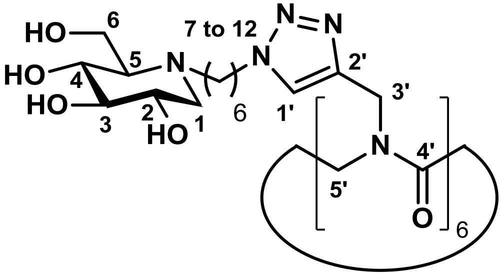

10a

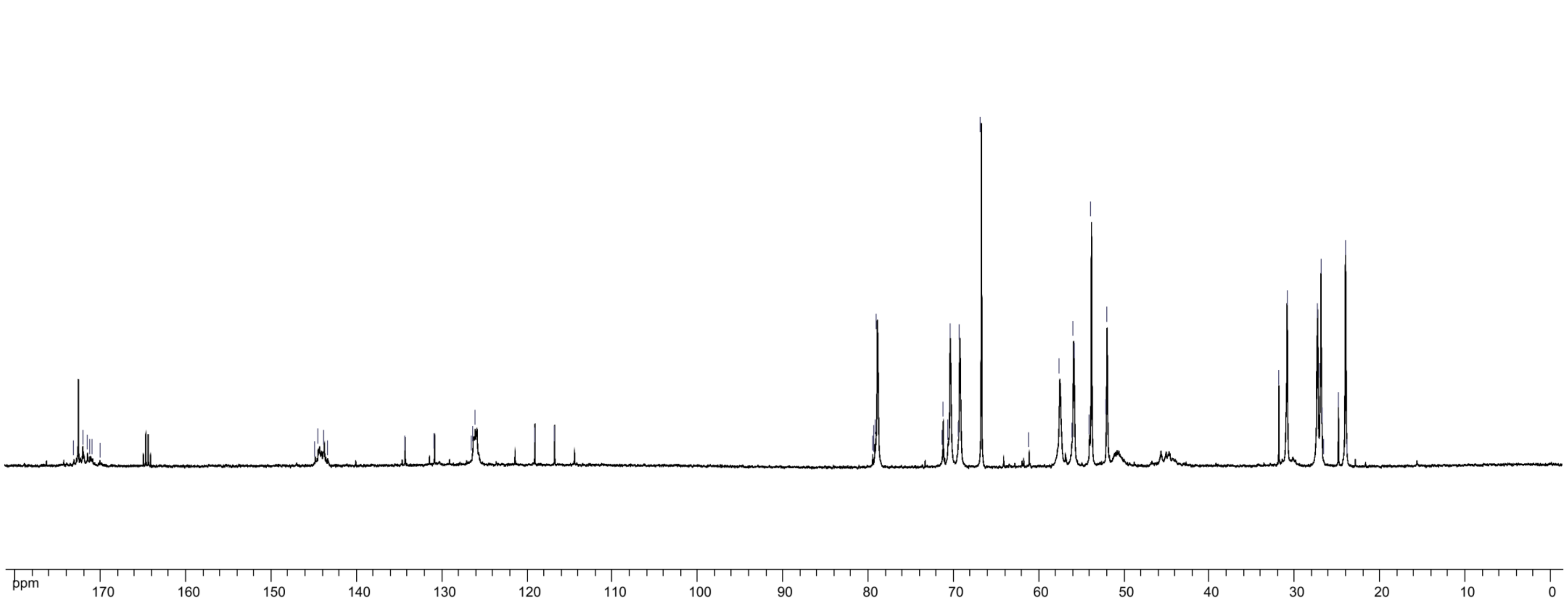

^{1}H NMR spectrum (D_2O/CD_3OD 1:1, 400 MHz) for compound 10b:

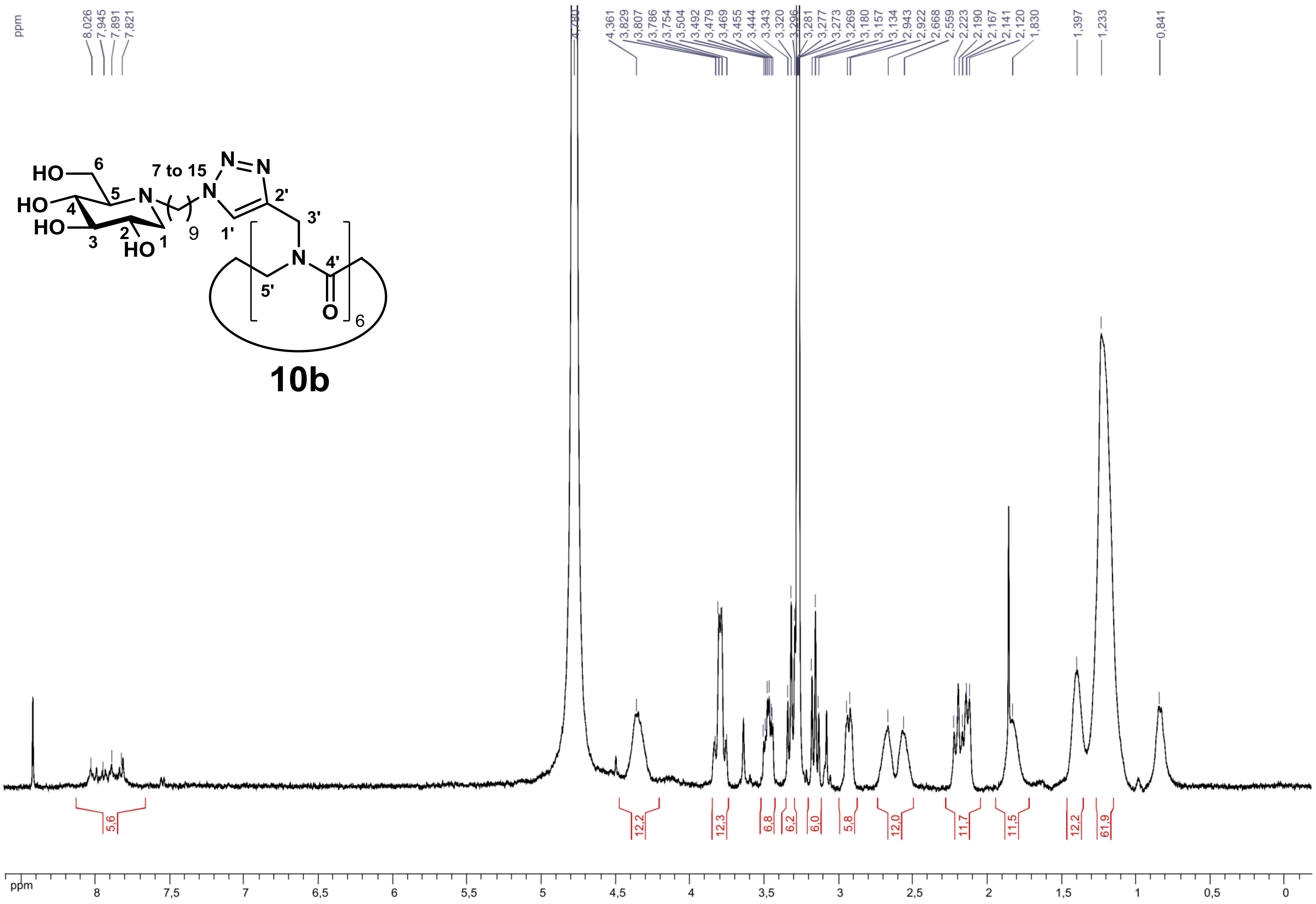

^{13}C NMR spectrum (D_2O + 0.1% TFA, 125 MHz) for compound 10b:

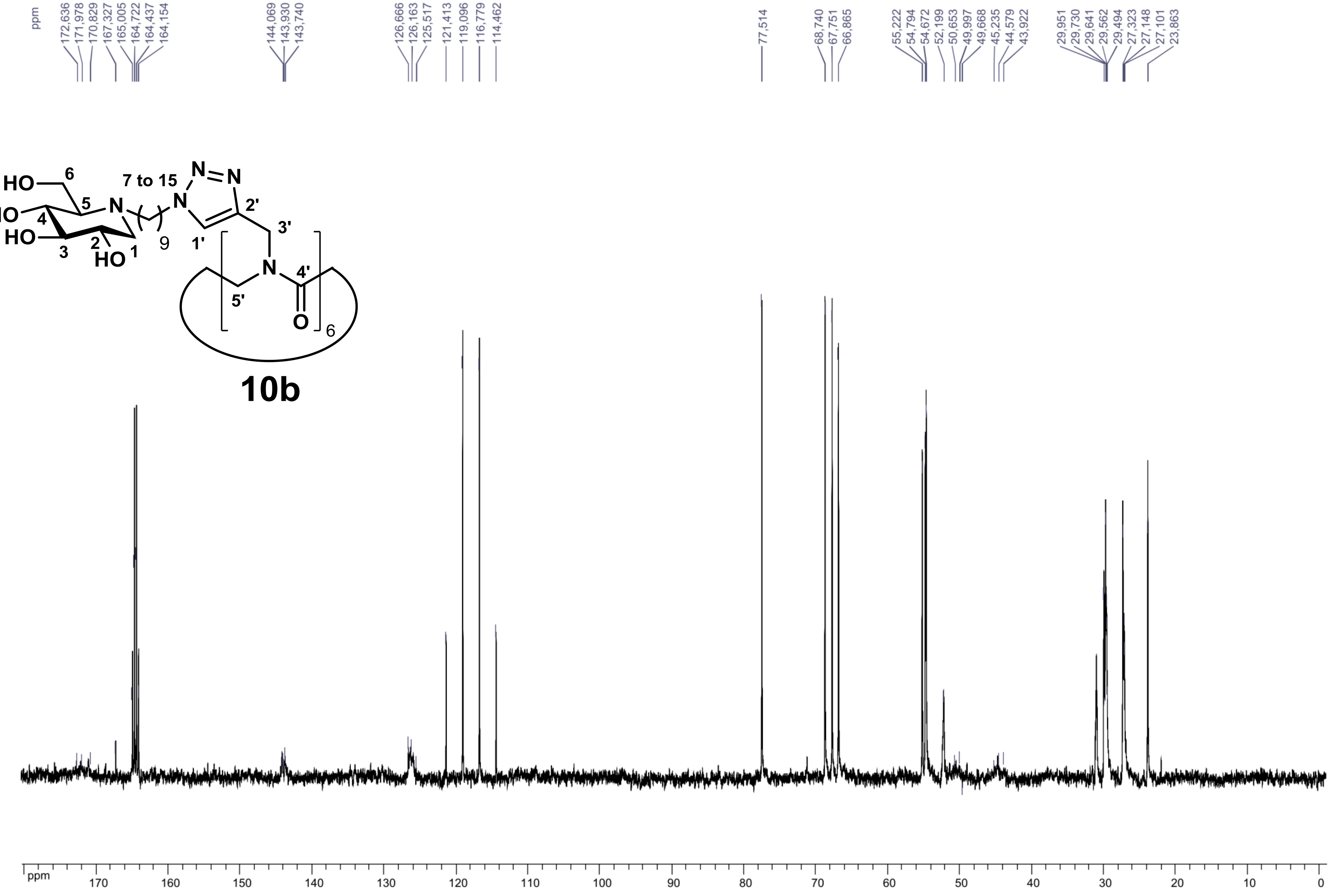

^{1}H NMR spectrum (D_2O/CD_3OD 1:1, 400 MHz) for compound 10c:

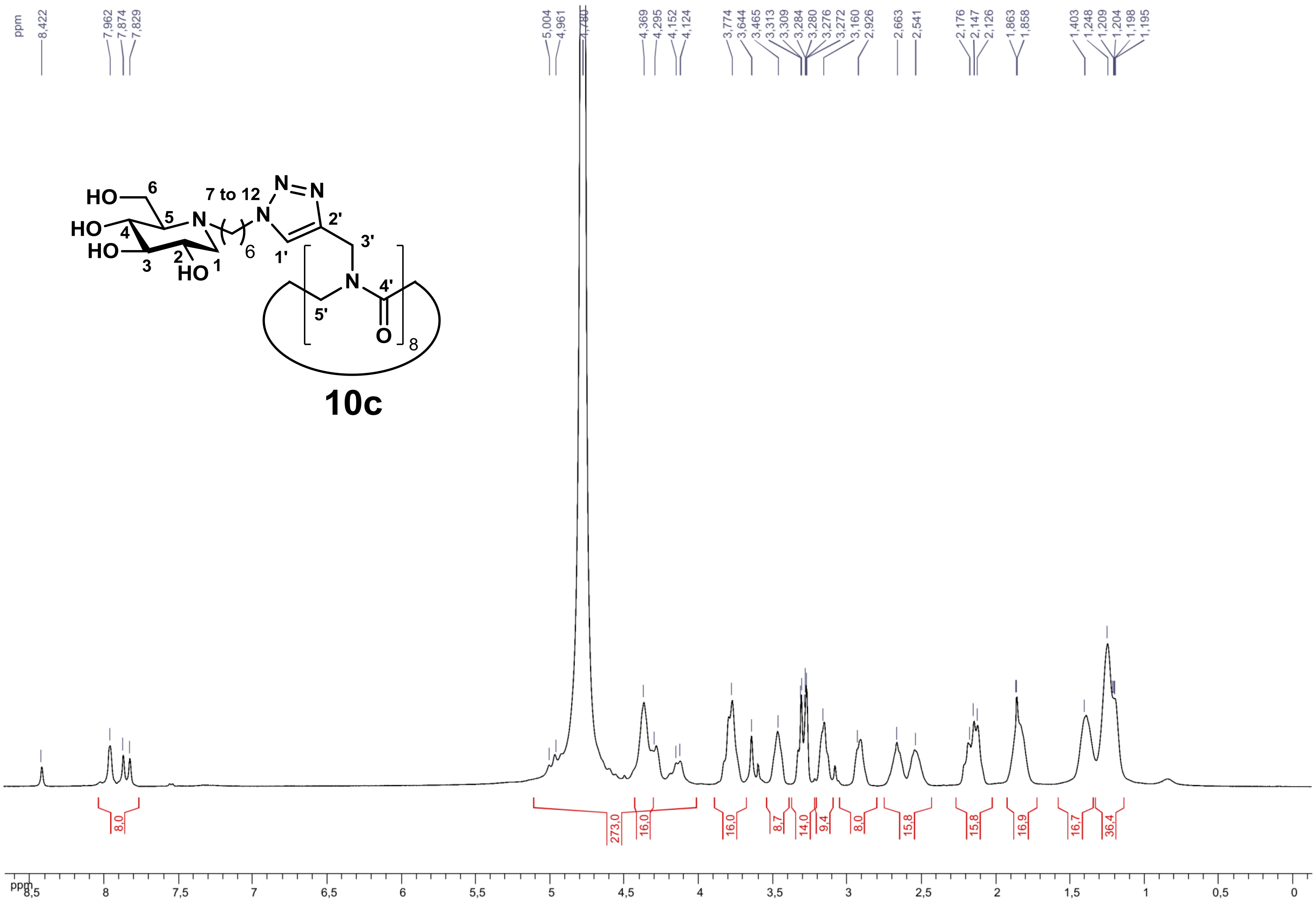

^{13}C NMR spectrum (D_2O/CD_3OD 1:1, 100 MHz) for compound 10c:

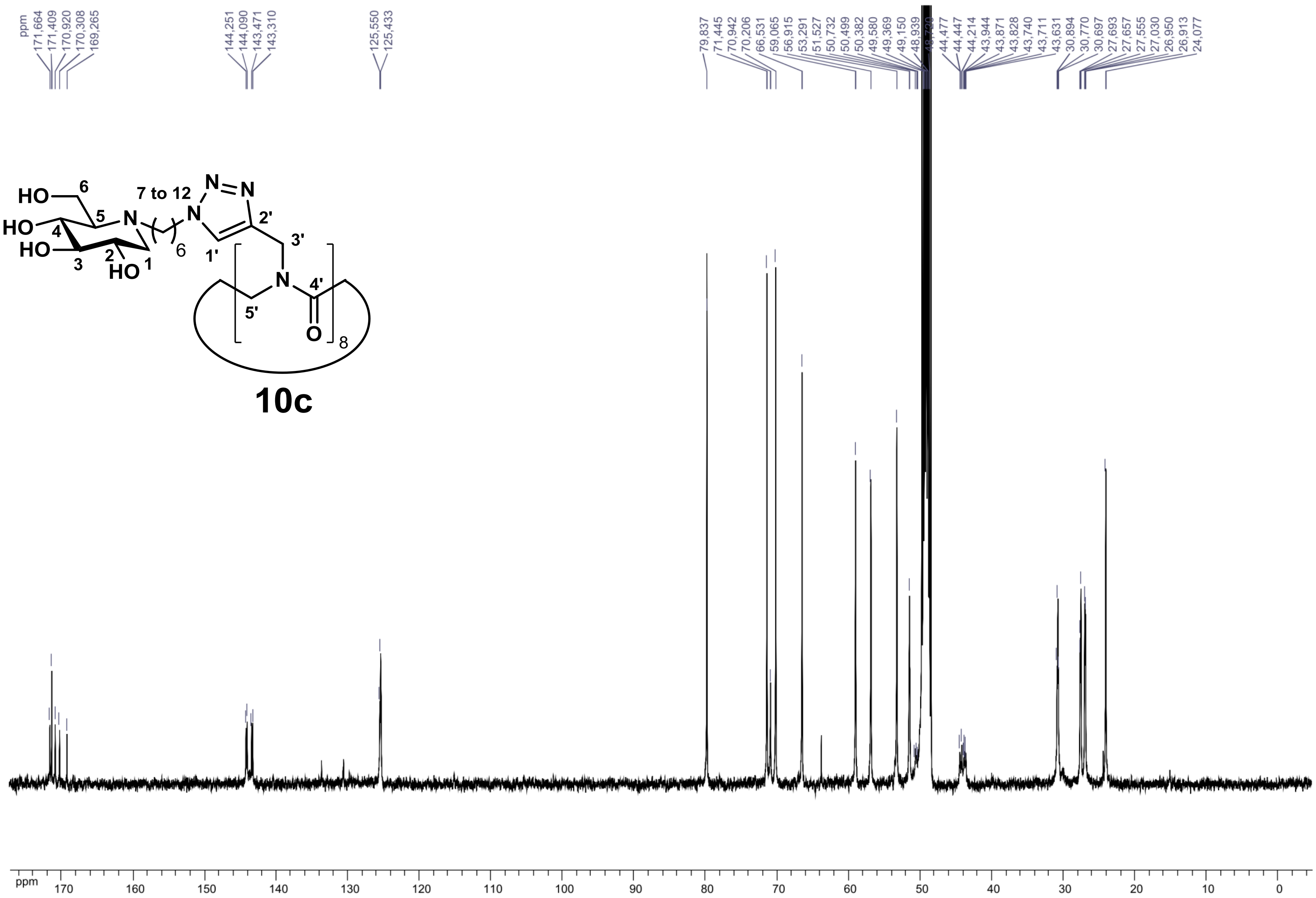

^{1}H NMR spectrum (CD_3OD, 400 MHz) for compound 10d:

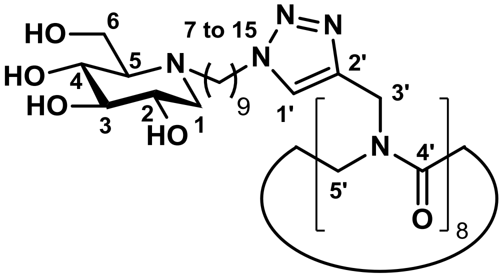

10d

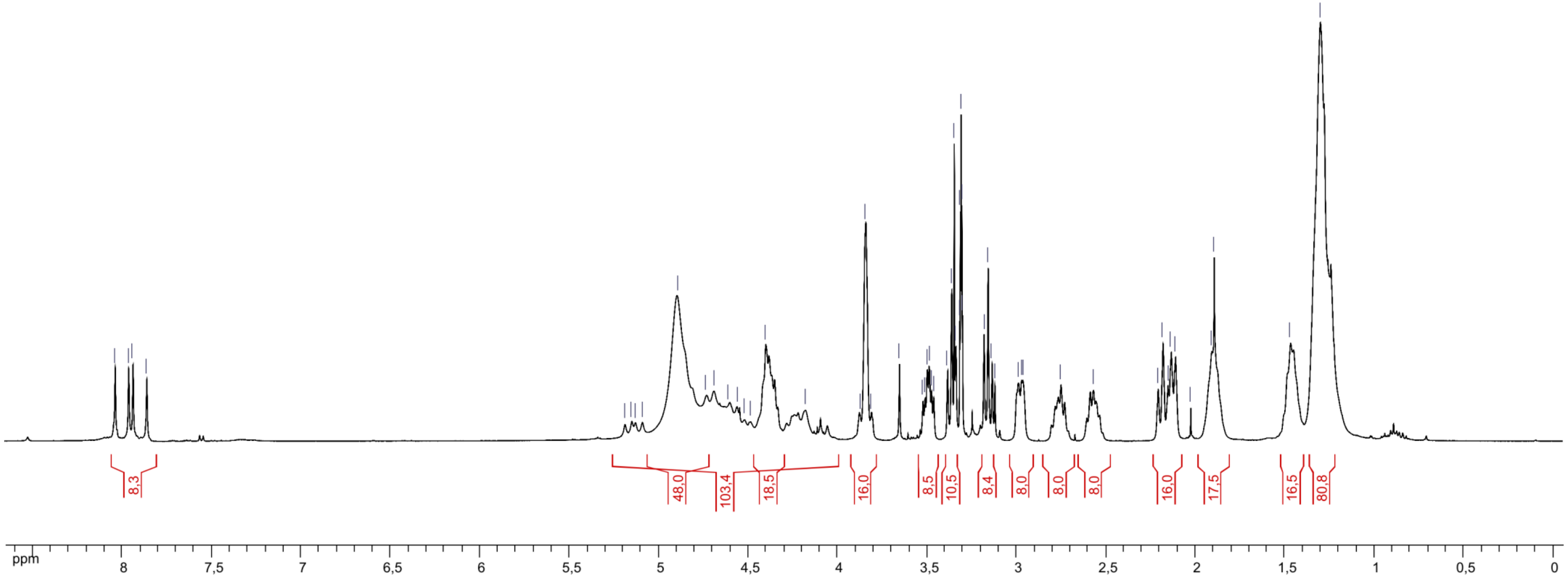

^{13}C NMR spectrum (CD_3OD, 100 MHz) for compound 10d:

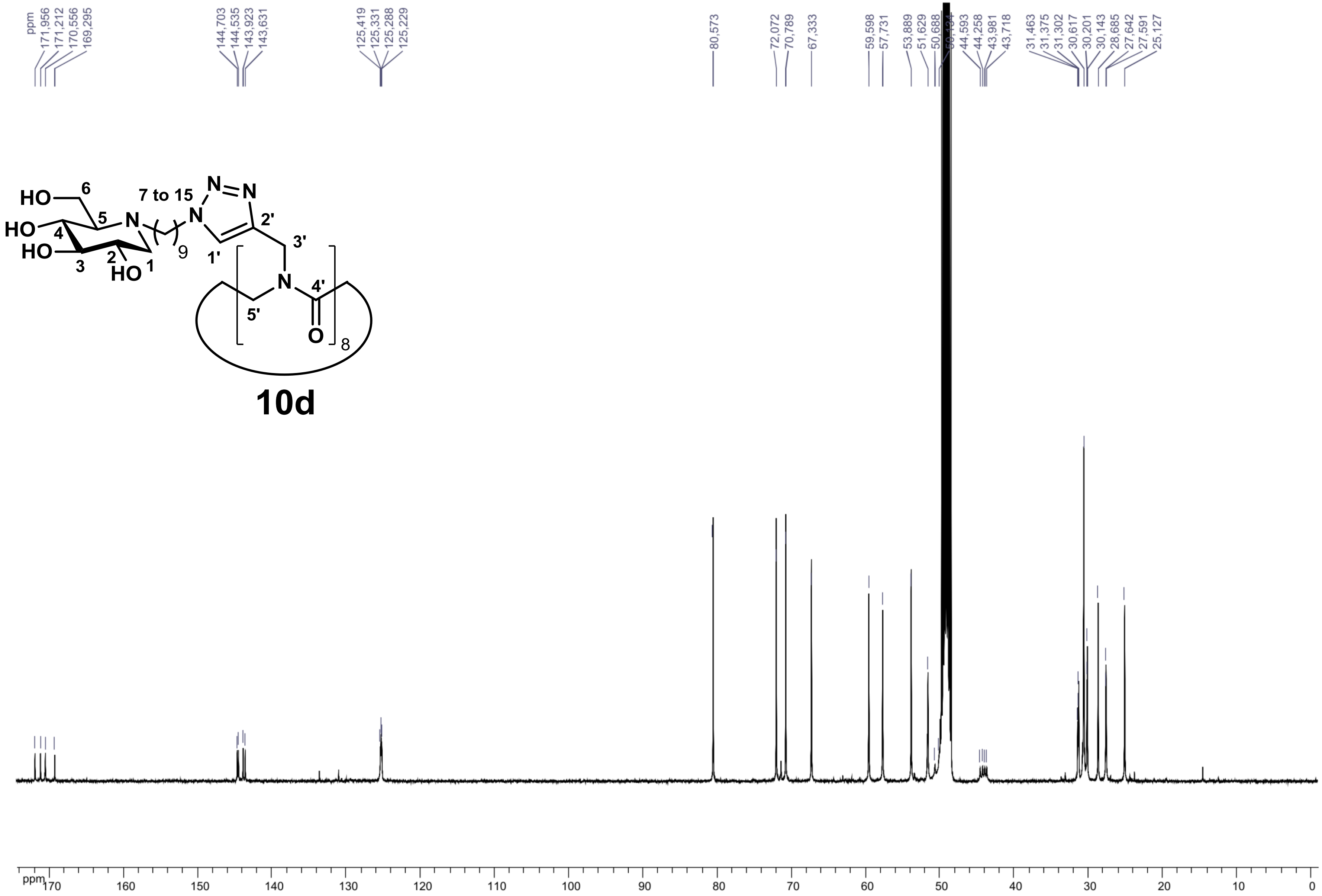

^{1}H NMR spectrum (D_2O, 500 MHz) for compound 10e:

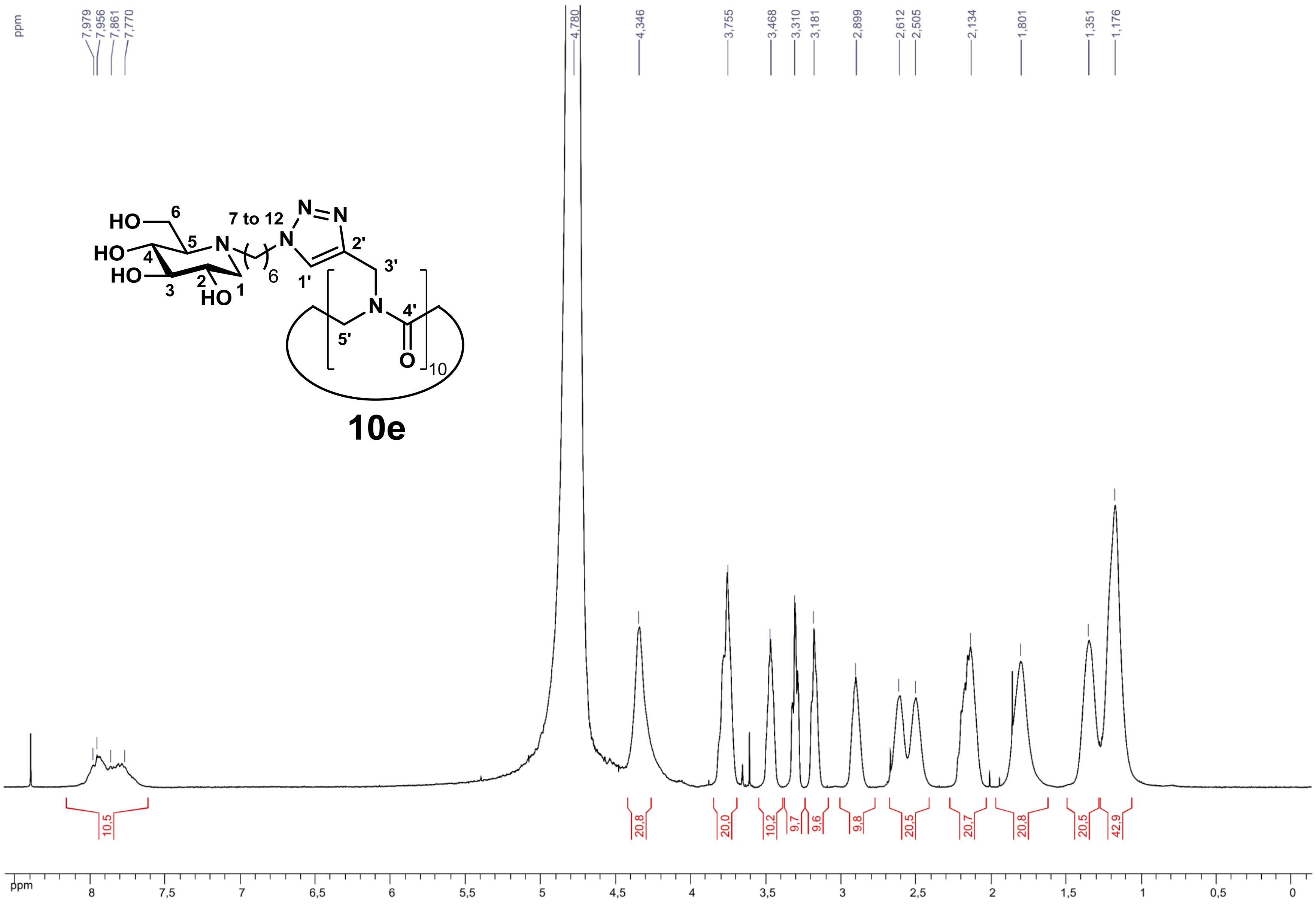

^{13}C NMR spectrum (D_2O, 125 MHz) for compound 10e:

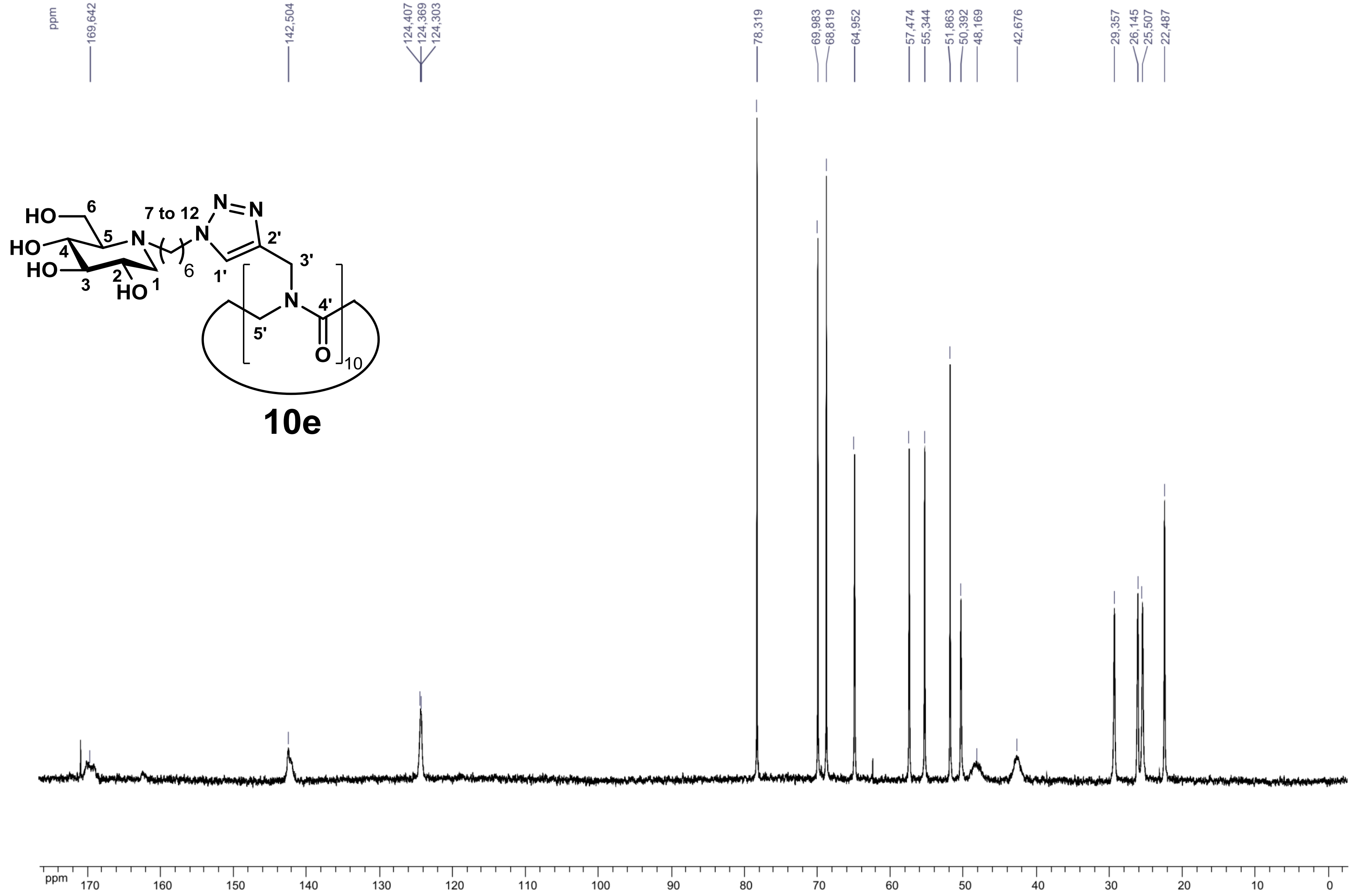

^{1}H NMR spectrum (D_2O/CD_3OD 1:1, 400 MHz) for compound 10f:

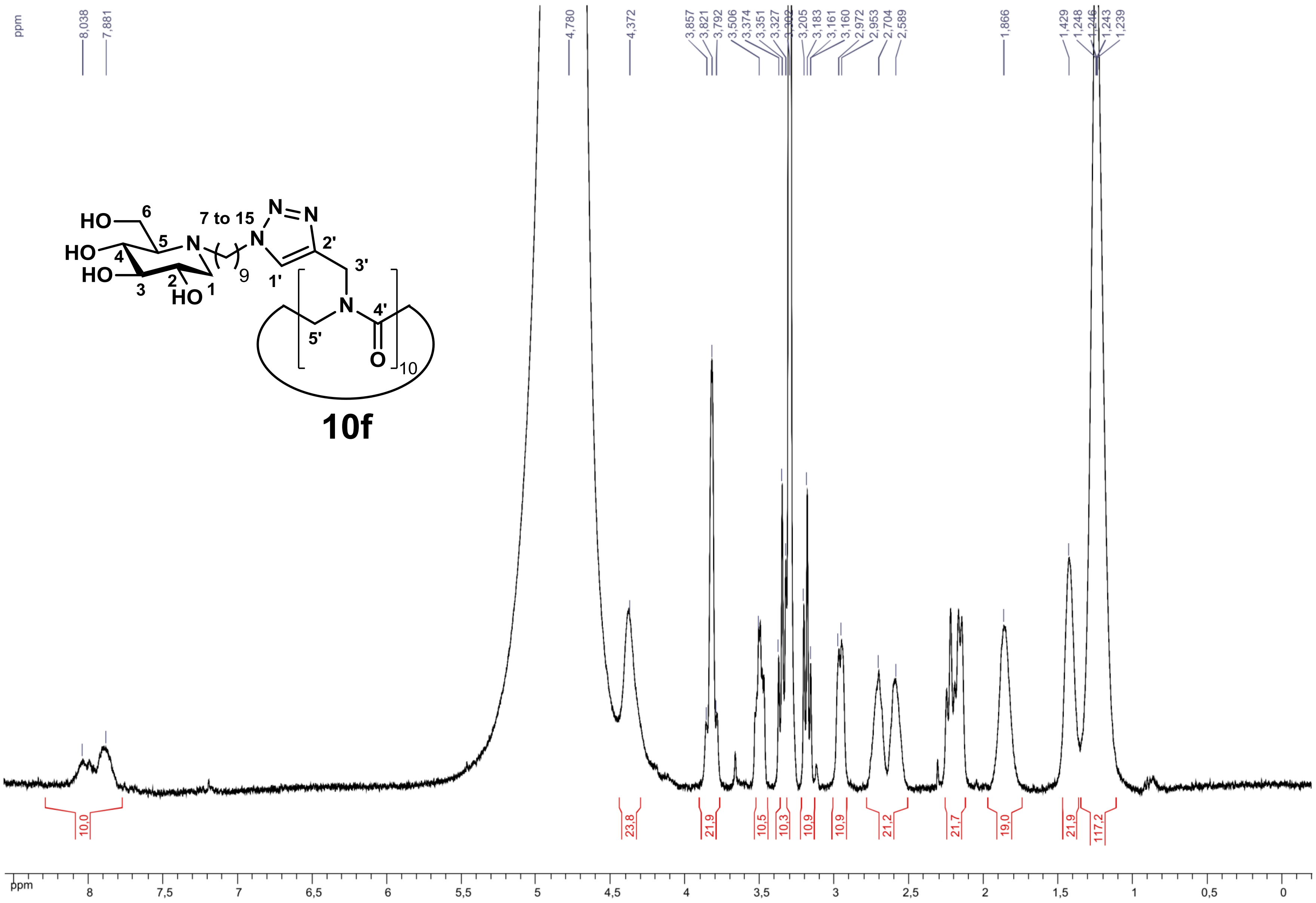

^{13}C NMR spectrum (CD_3CN/CD_3OD 1:1, 100 MHz) for compound 10f:

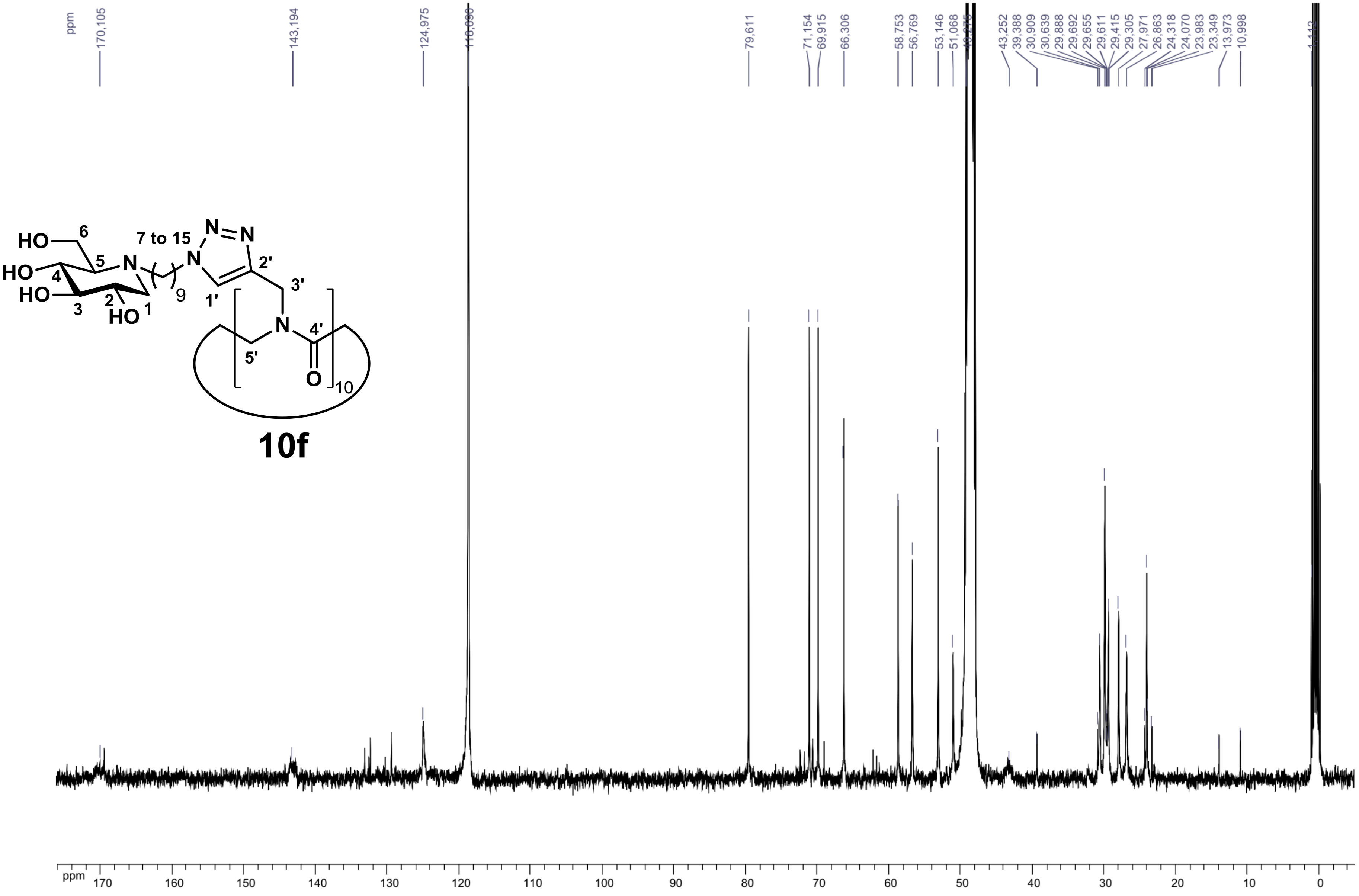

Supplement: File 1 — Mannosidase inhibition assay procedures, synthesis and NMR spectra of all new compounds. [file Beilstein_J_Org_Chem-10-1406-s001.pdf]
